# Efficacy and safety of a 4-week course of repeated subcutaneous ketamine injections for treatment-resistant depression (KADS study): randomised double-blind active-controlled trial

**DOI:** 10.1192/bjp.2023.79

**Published:** 2023-12

**Authors:** Colleen Loo, Nick Glozier, David Barton, Bernhard T. Baune, Natalie T. Mills, Paul Fitzgerald, Paul Glue, Shanthi Sarma, Veronica Galvez-Ortiz, Dusan Hadzi-Pavlovic, Angelo Alonzo, Vanessa Dong, Donel Martin, Stevan Nikolin, Philip B. Mitchell, Michael Berk, Gregory Carter, Maree Hackett, John Leyden, Sean Hood, Andrew A. Somogyi, Kyle Lapidus, Elizabeth Stratton, Kirsten Gainsford, Deepak Garg, Nicollette L. R. Thornton, Célia Fourrier, Karyn Richardson, Demi Rozakis, Anish Scaria, Cathrine Mihalopoulos, Mary Lou Chatterton, William M. McDonald, Philip Boyce, Paul E. Holtzheimer, F. Andrew Kozel, Patricio Riva-Posse, Anthony Rodgers

**Affiliations:** Black Dog Institute, University of New South Wales, Randwick, New South Wales, Australia; and George Institute for Global Health, Newtown, New South Wales, Australia; Central Clinical School, Faculty of Medicine and Health, University of Sydney, Sydney, New South Wales, Australia; and Australian Research Council Centre of Excellence for Children and Families over the Life Course, University of Sydney, Sydney, New South Wales, Australia; Australian Centre for Heart Health, Royal Melbourne Hospital, North Melbourne, Victoria, Australia; and NeuroCentrix, South Carlton, Victoria, Australia; Department of Psychiatry, University of Münster, Münster, Germany; Department of Psychiatry, Melbourne Medical School, University of Melbourne, Melbourne, Victoria, Australia; and Florey Institute of Neuroscience and Mental Health, Parkville, Victoria, Australia; Discipline of Psychiatry, University of Adelaide, Adelaide, South Australia, Australia; Australian National University School of Medicine and Psychology, Canberra, Australian Capital Territory, Australia; Dunedin School of Medicine, University of Otago, Dunedin, New Zealand; Mental Health and Specialist Services, Gold Coast Health, Bond University, Robina, Queensland, Australia; Department of Psychiatry and Mental Health, Hospital Universitari Parc Tauli, Sabadell, Spain; and Institut Investigacio I Innovacio Parc Tauli, Sabadell, Spain; Discipline of Psychiatry and Mental Health, University of New South Wales, Sydney, New South Wales, Australia; Discipline of Psychiatry and Mental Health, University of New South Wales, Sydney, New South Wales, Australia; University of New South Wales, Randwick, New South Wales, Australia; and George Institute for Global Health, Newtown, New South Wales, Australia; Institute for Mental and Physical Health and Clinical Translation (IMPACT), School of Medicine, Barwon Health, Deakin University, Geelong, Australia; College of Health, Medicine and Wellbeing, School of Medicine and Public Health, University of Newcastle, Callaghan, New South Wales, Australia; George Institute for Global Health, Newtown, New South Wales, Australia; Royal North Shore Hospital, St Leonards, New South Wales, Australia; and Northern Sydney Anaesthetic Research Institute, St Leonards, New South Wales, Australia; Division of Psychiatry, University of Western Australia, Perth, Western Australia, Australia; Discipline of Pharmacology, School of Biomedicine, Faculty of Health and Medical Sciences, University of Adelaide, Adelaide, Australia; Affective Care, Northwell Health, New York, New York, USA; Faculty of Medicine and Health, Central Clinical School, University of Sydney, Sydney, New South Wales, Australia; Epworth Centre for Innovation in Mental Health, Epworth Healthcare and Monash University, Camberwell, Victoria, Australia; Discipline of Psychiatry, University of Adelaide, Adelaide, South Australia, Australia; and Lysosomal Health in Ageing, Hopwood Centre for Neurobiology, South Australian Health and Medical Research Institute, Adelaide, South Australia, Australia; BrainPark, Turner Institute for Brain and Mental Health, Monash University, Clayton, Victoria, Australia; and Epworth Centre for Innovation in Mental Health, Epworth Healthcare and Monash University, Camberwell, Victoria, Australia; NeuroCentrix, Noble Park, Victoria, Australia; School of Public Health and Preventive Medicine, Monash University, Melbourne, Victoria, Australia; and School of Health and Social Development, Deakin University, Geelong, Australia; School of Public Health and Preventive Medicine, Monash University, Melbourne, Victoria, Australia; Department of Psychiatry and Behavioral Sciences, Emory University School of Medicine, Atlanta, Georgia, USA; Specialty of Psychiatry, Westmead Institute of Medical Research, Faculty of Medicine and Health, University of Sydney, Sydney, New South Wales, Australia; Department of Psychiatry, Geisel School of Medicine at Dartmouth, Lebanon, New Hampshire, USA; and Department of Surgery, Geisel School of Medicine at Dartmouth, Lebanon, New Hampshire, USA; Department of Behavioral Sciences and Social Medicine, Florida State University College of Medicine, Tallahassee, Florida, USA

**Keywords:** Ketamine/esketamine, major depressive disorder, clinical drug studies, neuroscience, affective disorders.

## Abstract

**Background:**

Prior trials suggest that intravenous racemic ketamine is a highly effective for treatment-resistant depression (TRD), but phase 3 trials of racemic ketamine are needed.

**Aims:**

To assess the acute efficacy and safety of a 4-week course of subcutaneous racemic ketamine in participants with TRD. Trial registration: ACTRN12616001096448 at www.anzctr.org.au.

**Method:**

This phase 3, double-blind, randomised, active-controlled multicentre trial was conducted at seven mood disorders centres in Australia and New Zealand. Participants received twice-weekly subcutaneous racemic ketamine or midazolam for 4 weeks. Initially, the trial tested fixed-dose ketamine 0.5 mg/kg versus midazolam 0.025 mg/kg (cohort 1). Dosing was revised, after a Data Safety Monitoring Board recommendation, to flexible-dose ketamine 0.5–0.9 mg/kg or midazolam 0.025–0.045 mg/kg, with response-guided dosing increments (cohort 2). The primary outcome was remission (Montgomery-Åsberg Rating Scale for Depression score ≤10) at the end of week 4.

**Results:**

The final analysis (those who received at least one treatment) comprised 68 in cohort 1 (fixed-dose), 106 in cohort 2 (flexible-dose). Ketamine was more efficacious than midazolam in cohort 2 (remission rate 19.6% *v*. 2.0%; OR = 12.1, 95% CI 2.1–69.2, *P* = 0.005), but not different in cohort 1 (remission rate 6.3% *v*. 8.8%; OR = 1.3, 95% CI 0.2–8.2, *P* = 0.76). Ketamine was well tolerated. Acute adverse effects (psychotomimetic, blood pressure increases) resolved within 2 h.

**Conclusions:**

Adequately dosed subcutaneous racemic ketamine was efficacious and safe in treating TRD over a 4-week treatment period. The subcutaneous route is practical and feasible.

Major depressive disorder is the second leading global cause of disability.^1^ Approximately one-third of people with major depression do not remit even after four trials of standard treatments.^2^ Failure to respond to two or more treatments is known as treatment-resistant depression (TRD). Ketamine is a novel, highly effective and rapidly acting treatment for TRD.^3^ The two main ketamine formulations and routes of administration in antidepressant trials to date have been intravenous infusions of racemic (R,S)-ketamine, a drug widely available in generic form, and a commercially developed intranasal spray containing S-ketamine (esketamine). Substantive phase 3 clinical trials (two of which met their primary end-points) have established the efficacy and safety of intranasal esketamine,^4–7^ which has regulatory approval for TRD in many countries. However, few randomised controlled trials (RCTs) have examined the efficacy and safety of repeated doses of racemic ketamine compared with a placebo/drug control.^8–12^ These trials reported promising results but were limited in sample size and underpowered (*n* = 5–81). The choice of comparator in trials has implications for final efficacy estimates. Most trials used a saline placebo, which, owing to lack of the psychotomimetic effects of ketamine, can lead to participant and treater unmasking.^13^ Recent studies have used an active control drug with psychoactive effects for better masking, for example midazolam.^14^ A meta-analysis found that five studies that compared ketamine with saline showed a much larger effect size (*d* = 1.8, 95% CI 1.4–2.2) than four studies that compared ketamine with midazolam (*d* = 0.7, 95% CI 0.4–0.9), measured the day after treatment.^15^

The optimal route of ketamine administration remains unclear. Racemic ketamine given by 40 min intravenous infusion has shown efficacy, but involves medical complexity and cost.^3^ Pilot RCTs giving racemic ketamine by intramuscular, subcutaneous, oral and intranasal routes^8–10,16^ suggest subcutaneous injection to be a relatively simple, safe and effective route.^16,17^ Finally, the safety of ketamine treatment has mostly been examined acutely for 2 h after each treatment, with fewer data on the cumulative, longer-term safety of repeated dosing.^18^ This trial examines the efficacy and safety of repeated racemic ketamine treatments over a 4-week period in adults with TRD, using subcutaneous administration. Midazolam was used as the active control. A structured framework comprehensively assessed acute and cumulative safety.

## Method

### Study design

The Ketamine for Adult Depression (KADS) study was a 4-week, randomised, double-blind, active-controlled, parallel-group, multicentre phase 3 trial recruited participants from six specialist mood disorders centres in Australia and one in New Zealand. The authors assert that all procedures contributing to this work comply with the ethical standards of the relevant national and institutional committees on human experimentation and with the Helsinki Declaration of 1975, as revised in 2008. All procedures involving human subjects/patients were approved by the Sydney Local Health District (RPAH Zone) Human Research Ethics Committee (Australia; X16-0146 and HREC/16/RPAH/168) and the Southern Health and Disability Ethics Committee (New Zealand; 16/STH/104). Participants voluntarily contacted the study team or were referred to the study by their doctor. All participants provided written informed consent and were assessed by a study doctor.

Main inclusion criteria were: age ≥18 years; major depressive disorder of at least 3 months’ duration, confirmed by the Structured Clinical Interview for DSM-5 Research Version; insufficient response to at least two adequate trials of antidepressant medications; any concurrent antidepressant medication at stable dosage ≥4 weeks prior to and during the RCT; and score ≥20 on the Montgomery–Åsberg Rating Scale for Depression (MADRS).^19^ For further details, see the Supplementary material, available at https://dx.doi.org/10.1192/bjp.2023.79, and the trial protocol, available at https://osf.io/6fpgu.

### Randomisation and masking(‘blinding’)

Participants were randomly assigned to receive racemic ketamine hydrochloride (100 mg/mL) or midazolam hydrochloride (5 mg/mL) in a 1:1 ratio. Both drugs were clear solutions for injection, prepared by a trial pharmacist, presented in vials of identical appearance. All study personnel (including the main statistician conducting analyses) were masked to the randomisation sequence, except for the trial statistician generating the sequence, the trial pharmacist and the Data Safety Monitoring Board members. Participants and raters were asked to guess their treatment allocation 3–4 days after the first treatment (pre-treatment at session 2) and after the last treatment at RCT end, and to provide reasons for their guess. See Supplementary material for further details.

### Procedures

Ketamine and midazolam were given subcutaneously into the abdominal wall twice per week for 4 weeks, with at least 3 days between treatments. The initial trial protocol (cohort 1) involved fixed doses at 0.5 mg/kg ketamine and 0.025 mg/kg midazolam (identical injection volume). At a routine Data Safety Monitoring Board meeting reviewing data from the first completed 51 participants, a revisiting of drug dosage was recommended as no participants in the entire masked sample had remitted and the safety profile was good. No interim analyses were planned or conducted. Thus, for cohort 2, flexible response-guided dosing was implemented. If participants had not improved by 50% from pretreatment baseline in MADRS scores at sessions 2, 4 and 6, dose escalation steps comprised 0.6, 0.75 and 0.9 mg/kg ketamine and 0.03, 0.0375 and 0.045 mg/kg midazolam (i.e. identical injection volumes between drugs at each dose level). After the 4-week treatment period, participants continued on any antidepressant medications that were established prior to study entry, with dosage unchanged prior to and during the trial, and were followed up 4 weeks later (RCT 4-week follow-up, at week 8). Those who had relapsed at this follow-up were eligible to enter an open-label treatment phase. See Supplementary material and the trial protocol for further details.

### Outcomes

The primary outcome was remission, defined as a MADRS score ≤10, assessed 3–4 days after the final treatment at the end of the 4-week RCT. Interrater reliability of MADRS raters was established by viewing and rating pre-recorded standardised interviews, with an intra-class correlation coefficient of >0.8 required against expert ratings. Structured assessments evaluated safety at each treatment session (acute effects), emerging or cumulative effects between sessions and changes from baseline to the RCT end and 4-week follow-up (long-term effects) using a prototype of the Ketamine Side Effect Tool (KSET)^20^ as well as other scales.

Key secondary outcomes over the RCT period were: remission based on a MADRS score ≤12 (for comparison with pivotal intranasal esketamine studies);^4,5^ response (50% improvement in MADRS score from RCT baseline); and change in MADRS scores from baseline to RCT end. Other secondary outcomes were MADRS outcomes at the post-RCT 4-week follow-up, and scores on Columbia Suicide Severity Rating Scale,^21^ Hamilton Anxiety Rating Scale,^22^ Clinical Global Impression-Severity scale (CGI-S) and Clinical Global Impression-Improvement scale (CGI-I),^23^ Health Economics Questionnaire and Assessment of Quality of Life 8 Dimensions (AQoL-8D).^24^ Outcomes other than MADRS and CGI scores will be reported elsewhere.

See Supplementary material and trial protocol for further details.

### Statistical analysis

The analyses used all participants who were randomised to a treatment arm and received at least one treatment (modified intention to treat).

The primary outcome was modelled using penalised logistic regression in the multiply imputed data-sets, with treatment, baseline MADRS score and site as covariates. A number needed to treat (NNT) was calculated from the difference in proportions with remission estimated from the multiply imputed data-sets. Heterogeneity of the primary outcome across cohorts was examined using logistic regression in the multiply imputed data-sets, with cohort, site, treatment arm, baseline MADRS score and treatment arm × cohort as covariates. Change in MADRS score from baseline to end of RCT was estimated from a linear mixed-effects model that included all baseline-to-session change scores as repeats (with an unstructured covariance matrix), and session, treatment, site, baseline MADRS, and the session × treatment and session × baseline MADRS interactions as covariates.

CGI-S and CGI-I scores were treated as ordinal categories and the odds of a lower severity or greater improvement for ketamine versus midazolam were modelled using ordinal regression (with baseline CGI-S and site as fixed factors). Further details are available in the Supplementary material; see https://osf.io/6fpgu for the full analysis plan, which was published prior to data analysis.

## Results

Recruitment started on 15 August 2016 and closed in April 2020 owing to COVID-19 restrictions. Follow-up was completed in May 2020 and data were extracted 1 July 2021. In total, 1033 individuals were assessed for eligibility and 184 were randomised. Three individuals withdrew consent for the use of their data, leaving 73 participants in cohort 1 (fixed-dose) and 108 in cohort 2 (flexible-dose), of whom 68 and 106 respectively received at least one allocated dose. Most participants received all eight doses (Supplementary material). Most participants in cohort 2 (30/53 ketamine, 38/53 midazolam) were escalated to the highest dose level (see CONSORT diagram in Supplementary material). Baseline clinical and demographic details of the randomised groups within each cohort are shown in Table 1.

**Table 1:** Baseline characteristics for the study sample^a^

	Cohort 1 (Fixed-dose)		Cohort 2 (Flexible-dose)	
	Midazolam	Ketamine	Midazolam	Ketamine
Randomised, *n*^b^	38	35	54	54
mITT, *n*	35	33	53	53
Age, years: mean (s.d.)	48.2 (13.6)	45.9 (11.5)	46.2 (15.3)	44.5 (13.6)
Female gender, *n* (%)	8 (22.9)	9 (27.3)	21 (39.6)	20 (37.7)
Body weight, kg: mean (s.d.)	85.8 (17.6)	95.1 (24.9)	85.4 (21.5)	87.6 (22.0)
Participants per site, *n* (%)				
Site 1	7 (20.0)	7 (21.2)	4 (7.5)	8 (15.1)
Site 2	0 (0.0)	0 (0.0)	8 (15.1)	6 (11.3)
Site 3	0 (0.0)	0 (0.0)	5 (9.4)	5 (9.4)
Site 4	4 (11.4)	4 (12.1)	4 (7.5)	3 (5.7)
Site 5	5 (14.3)	4 (12.1)	12 (22.6)	11 (20.8)
Site 6	3 (8.6)	3 (9.1)	10 (18.9)	12 (22.6)
Site 7	16 (45.7)	15 (45.5)	10 (18.9)	8 (15.1)
Age at onset of first major depressive episode, years: mean (s.d.)	24.7 (12.5)	24.0 (10.3)	24.5 (14.7)	25.1 (11.3)
Duration of current major depressive episode, years: mean (s.d.)	5.2 (6.7)	6.5 (8.7)	5.2 (8.0)	8.2 (8.8)
Number of unsuccessful antidepressant trials, mean (s.d.)				
Current episode	4.5 (3.5)	3.4	3.1 (2.4)	3.6 (2.2)
Total lifetime	6.4 (3.7)	5.8	5.6 (2.3)	5.4 (2.9)
ECT failed for current episode, *n* (%)	10 (28.6)	9	10 (18.9)	12 (22.6)
Concurrent psychotropic medications, *n* (%)				
None	7 (20.0)	3 (9.1)	7 (13.2)	5 (9.4)
Antidepressant	23 (65.7)	26 (78.8)	42 (79.2)	42 (79.2)
Antipsychotic	6 (17.1)	8 (24.2)	17 (32.1)	12 (22.6)
Lithium/anticonvulsant/ mood stabiliser	9 (25.7)	9 (27.3)	12 (22.6)	11 (20.8)
Benzodiazepine	6 (17.1)	11 (33.3)	20 (37.7)	15 (28.3)
Melancholia,^c^ *n* (%)	22 (62.9)	27 (81.8)	24 (45.3)	31 (58.5)
Baseline MADRS total score, mean (s.d.)	30.8 (6.3)	30.7 (4.8)	30.3 (5.2)	28.9 (5.7)
Baseline HAM-A total score, mean (s.d.)	20.7 (5.1)	21.9 (5.3)	20.0 (8.0)	21.0 (8.5)
Baseline C-SSRS score, mean (s.d.)	1.6 (1.4)	1.6 (1.4)	1.9 (1.4)	1.5 (1.3)

C-SSRS, Columbia Suicide Severity Rating Scale; ECT, electroconvulsive therapy; HAM-A, Hamilton Anxiety Rating Scale; MADRS, Montgomery–Åsberg Rating Scale for Depression; mITT, modified intention to treat.

a. Percentages reported are based on the mITT sample, i.e. participants who received at least one treatment.

b. Excludes 3 participants from the original 184 randomised who subsequently withdrew consent for use of their data.

c. Assessed by the Structured Clinical Interview for DSM-5 Research Version.

### Primary outcome

**Fig. 1:**
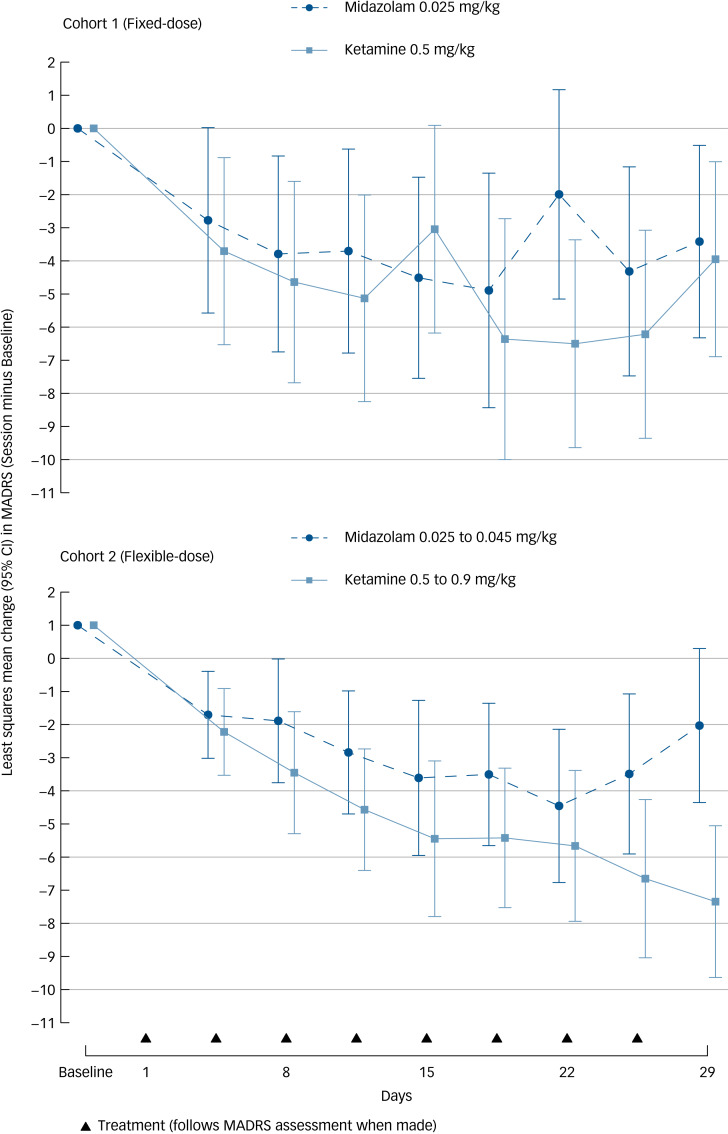
Change from baseline in Montgomery–Åsberg Rating Scale for Depression (MADRS) scores across the randomised controlled trial phase for the two cohorts.

For the primary outcome (remission defined as MADRS score ≤10), there was no statistically significant difference in remission rates between treatments for cohort 1 (fixed-dose) (OR = 1.34, 95% CI 0.22–8.21, *P* = 0.76; remission rates 6.3% for ketamine and 8.8% for midazolam). There was a significant difference between treatments for cohort 2 (flexible-dose) (remission rates 19.6% for ketamine, 2.0% for midazolam) favouring ketamine (OR = 12.11, 95% CI 2.12–69.17, *P* = 0.005) (NNT = 6.01, 95% CI 3.34–30.58) (Fig. 1).

The odds favouring ketamine in cohort 2 were higher than the odds in cohort 1 (OR = 12.96, 95% CI 1.10–152.54, *P* = 0.04). On the primary outcome measure, few data were lost at the end of RCT assessment (2/68 in cohort 1 and 6/106 in cohort 2), leaving limited scope for influence from multiple imputation. Remission and response rates in the two cohorts are presented in Table 2.

**Table 2:** Primary and key secondary efficacy outcomes at the trial end

	Cohort 1 (Fixed-dose)							Cohort 2 (Flexible-dose)						
	Midazolam		Ketamine		Treatment effect estimate			Midazolam		Ketamine		Treatment effect estimate		
Participants (mITT/End RCT^a^), *n*	35/34		33/32					53/49		53/51				
End RCT MADRS total score, mean (s.d.)	26.6 (9.6)		26.4 (9.3)					26.7 (7.5)		20.6 (9.9)				
					Ketamine versus Midazolam							Ketamine versus Midazolam		
	*n*	(%)	*n*	(%)	OR^b^	95% CI	*P*	*n*	(%)	*n*	(%)	OR^b^	95% CI	*P*
Remission MADRS ≤ 10	3	(8.8)	2	(6.3)	1.34	0.22 to 8.21	0.76	1	(2.0)	10	(19.6)	12.11	2.12 to 69.17	0.005
Remission MADRS ≤ 12	4	(11.8)	2	(6.3)	0.95	0.15 to 6.09	0.96	2	(4.1)	11	(21.6)	8.26	1.80 to 37.86	0.007
Response (MADRS change ≥ 50%)	3	(8.8)	3	(9.4)	2.20	0.36 to 13.33	0.39	2	(4.1)	15	(29.4)	12.25	2.71 to 55.44	0.001
					Ketamine versus Midazolam							Ketamine versus Midazolam		
	Mean	(s.d.)	Mean	(s.d.)	Δ	95%CI	*P*	Mean	(s.d.)	Mean	(s.d.)	Δ	95%CI	*P*
Change in MADRS (from baseline)	−3.94	(6.92)	−4.28	(6.39)	−0.53	−3.78 to 2.71	0.74	−3.45	(6.57)	−8.18	(10.2)	−5.45	−8.72 to −2.17	0.001

RCT, randomised controlled trial; MADRS, Montgomery–Åsberg Rating Scale for Depression; mITT, modified intention to treat, i.e. participants who received at least one treatment.

a. For End RCT, *n* reflects the participants with a MADRS score available at that time point.

b. Odds ratio (OR) from multiply imputed data-sets, adjusted for covariates. An OR > 1 favours ketamine.

The Supplementary material gives pre-specified sensitivity, heterogeneity and subgroup analyses. The key findings of note were a signal of greater treatment effects (after ketamine treatment compared with midazolam) in those with higher baseline anxiety scores, those with a greater number of prior failed antidepressant treatments and those taking a concomitant antipsychotic.

### Secondary outcomes

For change in mean MADRS scores, ≥50% reduction in MADRS scores (i.e. response) and remission (defined as MADRS score ≤12), there was no significant difference between groups in cohort 1 at the end of the RCT. However, in cohort 2 the reduction in mean MADRS score by treatment end differed between groups by 5.5 points (95% CI 2.1–8.7), and response rates (29% *v*. 4%, *P* = 0.001) and remission rates (MADRS ≤ 12) (22% *v*. 4%, *P* = 0.007) were greater, favouring the ketamine group. Similarly, CGI-S and CGI-I scores showed no between-group differences in cohort 1, but significantly better outcomes in the ketamine group in cohort 2 (Supplementary material).

### Post-RCT follow-up

At 4 weeks after the last treatment, the difference in remission rates between ketamine and midazolam in cohort 2 was reduced (8.0% for ketamine, 2.1% for midazolam; OR = 2.02, 95% CI 0.40–10.28, *P* = 0.4). No between-group differences in MADRS scores were statistically significant for remission (MADRS ≤10 or MADRS ≤12), response or change in mean scores from baseline in either cohort (Supplementary material). For CGI-S and CGI- I scores, significant between-group differences were seen only in cohort 1, favouring midazolam (Supplementary material). Few follow-up data are available at 8 weeks and 6 months after RCT as most participants proceeded to open label treatment after the RCT 4-week follow-up. These data are presented in the Supplementary material.

### Safety outcomes

Serious adverse events were rare and most were unrelated to the study drug: in cohort 1 there were two in the midazolam group (suicide attempt (unrelated) and mood deterioration (unrelated) and none in the ketamine group; in cohort 2 there were three in the midazolam group (suicide attempt (unrelated), increased suicidal ideation (unrelated) and wrist injury (unrelated)) and two in the ketamine group (major dissociative episode (related) and auditory hallucination (related)). There were no deaths reported throughout the study. Four ketamine participants (*n* = 2 in each in cohort) experienced adverse events (non-serious) which led to discontinuation of intervention during the RCT phase (skin rash, increased anxiety, headache, increased depression). The well-established acute effects of ketamine (psychotomimetic, blood pressure elevation, etc.) were observed in both cohorts, being greater in cohort 2 (Table 3). These acute effects resolved or returned to pretreatment levels within the 2 h observation period. No participants required medical intervention. See Supplementary material for further details. There was no evidence of cognitive impairment in either treatment group in either cohort (details to be reported elsewhere).

**Table 3:** Acute post-treatment adverse events (over the first 60 min post dosing)^a^

	Cohort 1 (Fixed-dose)					Cohort 2 (Flexible-dose)				
	Midazolam (*n* = 35)		Ketamine (*n* = 33)			Midazolam (*n* = 53)		Ketamine (*n* = 53)		
Adverse event	*n*	(%)	*n*	(%)	*P*	*n*	(%)	*n*	(%)	*P*
Sedation	29	(82.9)	29	(87.9)	0.74	50	(94.3)	45	(84.9)	0.20
Light-headedness	10	(28.6)	20	(60.6)	0.01	14	(26.4)	42	(79.2)	<0.001
Reduced concentration	9	(25.7)	18	(54.5)	0.03	18	(34.0)	39	(73.6)	<0.001
Dissociation^b^	4	(11.4)	16	(48.5)	0.001	13	(24.5)	41	(77.4)	<0.001
Dry mouth	8	(22.9)	14	(42.4)	0.12	18	(34.0)	30	(56.6)	0.03
Numbness/tingling	3	(8.6)	18	(54.5)	<0.001	8	(15.1)	39	(73.6)	<0.001
Dizziness/vertigo	4	(11.4)	15	(45.5)	0.003	9	(17.0)	38	(71.7)	<0.001
Weakness/fatigue	8	(22.9)	17	(51.5)	0.02	9	(17.0)	27	(50.9)	<0.001
Confusion	0	(0.0)	14	(42.4)	<0.001	8	(15.1)	33	(62.3)	<0.001
Visual changes	5	(14.3)	15	(45.5)	0.007	4	(7.5)	29	(54.7)	<0.001
Dysgeusia	5	(14.3)	9	(27.3)	0.24	8	(15.1)	20	(37.7)	0.02
Restlessness	3	(8.6)	7	(21.2)	0.18	10	(18.9)	21	(39.6)	0.02
Feeling hot/cold or sweating	8	(22.9)	8	(24.2)	1.00	4	(7.5)	18	(34.0)	0.001
Headache/pressure in head	7	(20.0)	8	(24.2)	0.77	8	(15.1)	14	(26.4)	0.23
Anxiety	1	(2.9)	10	(30.3)	0.003	7	(13.2)	16	(30.2)	0.06
Tinnitus	2	(5.7)	9	(27.3)	0.02	3	(5.7)	13	(24.5)	0.013
Chest heaviness/discomfort	3	(8.6)	5	(15.2)	0.47	2	(3.8)	16	(30.2)	<0.001
Abnormal movements	3	(8.6)	7	(21.2)	0.18	2	(3.8)	13	(24.5)	0.002
Moodiness	0	(0.0)	7	(21.2)	0.004	2	(3.8)	14	(26.4)	0.002
Nausea	2	(5.7)	6	(18.2)	0.14	3	(5.7)	10	(18.9)	0.07
Shortness of breath	1	(2.9)	6	(18.2)	0.05	3	(5.7)	8	(15.1)	0.20
Hypersalivation	0	(0.0)	4	(12.1)	0.05	1	(1.9)	9	(17.0)	0.02
Palpitations	0	(0.0)	3	(9.1)	0.11	5	(9.4)	5	(9.4)	1.00
Euphoria^c^	1	(2.9)	1	(3.0)	1.00	0	(0.0)	8	(15.1)	0.006
Relaxation/reduced anxiety	0	(0.0)	1	(3.0)	0.49	4	(7.5)	4	(7.5)	1.00
Skin changes (rash, itch)	2	(5.7)	1	(3.0)	1.00	1	(1.9)	3	(5.7)	0.62
Heaviness in limbs/body	0	(0.0)	1	(3.0)	0.49	0	(0.0)	3	(5.7)	0.24
Altered/increased perception	1	(2.9)	3	(9.1)	0.35	0	(0.0)	0	(0.0)	1.00
Depersonalisation	0	(0.0)	2	(6.1)	0.23	0	(0.0)	1	(1.9)	1.00
Mood deterioration	0	(0.0)	2	(6.1)	0.23	0	(0.0)	0	(0.0)	1.00

a. Adverse events were evaluated using the Clinician-Administered Dissociative States Scale (CADSS), the Young Mania Rating Scale (YMRS) and Section B of the Ketamine Side Effect Tool (KSET). Section B of the KSET reflects symptoms observed at any time in the 60 min following a ketamine or midazolam injection. The incidence of participants experiencing each adverse event was compared between ketamine and midazolam groups using a Fisher's exact test. Events are reported in order from highest to lowest frequency across all participants.

b. This includes subjective reports of dissociation as well as the number of participants with a >4-point increase in the CADSS total score from baseline.

c. Euphoria was operationalised as the number of participants with a score of ≥4 on item 1 of the YMRS.

### Masking of treatment assignment at the time of primary outcome assessment

Participant and rater guesses of treatment allocation, made at the time of primary outcome assessment, are judged as most relevant to interpretation of the primary outcome. Bang Blinding Index scores (Supplementary material) in cohort 1 were statistically significantly positive (i.e. a tendency for guesses to be correct) for both participants and raters, for midazolam but not ketamine. In cohort 2, masking was not achieved for raters or participants in the midazolam group, and was achieved for raters but not participants in the ketamine group. Across treatment groups in both cohorts, most rater guesses (90–100%) were based on treatment efficacy, whereas most participant guesses were based on treatment efficacy or a combination of efficacy and treatment experience (Supplementary material). See Supplementary material for further details.

## Discussion

This was the largest randomised controlled trial of racemic ketamine tested against placebo in participants with TRD, with cohort 2 alone being larger than total samples in previous trials. It showed the efficacy and safety of an adequately dosed 4-week treatment course given by subcutaneous injection, tested against an active control drug. The superior antidepressant efficacy of ketamine was evident only in cohort 2 (flexible-dose), which involved response-guided dose escalation from 0.5 mg/kg up to 0.9 mg/kg. However, it was not evident in cohort 1 (fixed-dose), which involved a dose of 0.5 mg/kg for all treatments. In this severely treatment-resistant population, of which 24% had failed to respond to treatment with electroconvulsive therapy, adequately dosed racemic ketamine produced benefits that were large, being both clinically and statistically superior to midazolam.

### Limitations

There are several important limitations to the trial. First, the original dosing protocol was changed on the advice of the Data Safety Monitoring Board based on results of the first 51 completers, owing to lack of efficacy. Data indicating the bioavailability of subcutaneous ketamine to be about 0.66 were not available when the present study was designed,^25^ and they suggest that 0.75 mg/kg subcutaneously is required to approximate 0.5 mg/kg given by intravenous infusion, which was shown to be effective in prior trials.^3^ Although this mid-study adjustment provided useful insights on two approaches to dosing, it meant that neither cohort achieved the originally planned sample size. Study recruitment was affected by the COVID-19 pandemic, being halted in April 2020 and with significant challenges in restarting. In addition, the study was designed and funded to examine the effects of a 4-week treatment period, with follow-up of progress over a further 4 weeks. It was not designed to assess the longer-term effects of continued ketamine administration, an important question for future examination. Such studies have been informative for esketamine therapy, with efficacy and safety maintained over 1 year among those who initially responded.^4,26^ The current results do confirm that if ketamine treatment is halted after 4 weeks, the benefits are not sustained for all remitters and that ongoing treatment should be considered.

As with phase 3 trials of esketamine, final ratings were conducted by independent raters masked to treatment allocation. However, in contrast to the trials of esketamine, efficacy and safety were tested against an active control drug with psychoactive effects to facilitate masking, and there was a formal evaluation of masking (which remains the exception rather than the norm in trials in this area and in psychiatry trials more broadly).^13,27^ Our results indicate that masking was not completely achieved, particularly for participants, despite the use of an active control. This is likely to be an issue for all studies involving subanaesthetic doses of ketamine, and it may be that masking cannot be completely achieved, given the characteristic subjective effects of ketamine, and this may have influenced measured outcomes in this and other studies in this field. The degree to which unmasking, with attendant expectancy and disappointment effects, is at least partially an explanation for the benefits seen in cohort 2 remains uncertain, but several lines of evidence indicate that it does not account for all the effect. Both raters and participants indicated that their guesses were more related to treatment efficacy than treatment experience, suggesting their guesses were influenced by post-treatment changes in mood (or lack of them) more than intra-treatment subjective effects. Further, there was statistically significant unmasking in cohort 1 but no significant treatment effect. Finally, use of ketamine intraoperatively under general anaesthesia also confers reductions in postoperative mood scores to a similar amount as that seen in cohort 2 of this trial.^28^ Future studies should assess and report masking, but this should be considered in the context that any effective treatment for a patient-reported outcome such as depression will be unmasked once that treatment's effect is established, even if the treatment administration is perfectly masked.

### Significance for the field

This study addresses several important gaps in the literature. To date, substantive studies of efficacy and safety in TRD are available for intranasal esketamine but not racemic ketamine. The largest previous RCT of racemic ketamine, involving 81 participants,^8^ was in non-treatment-resistant depression. Other studies were small proof-of-concept studies piloting treatment technique or dosing approaches and often of a single treatment only.^9–12^ Only one used a control with psychoactive effects.^10^

In this study, adequately dosed racemic ketamine was shown to have superior antidepressant efficacy to an active control drug, with the proportional increase in remission comparing favourably to that of studies of intranasal esketamine tested against a non-active placebo.^5^ This is despite participants in this study having a higher level of treatment resistance, with 24% having failed to respond to electroconvulsive therapy (an exclusion criterion in intranasal esketamine studies),^5,6^ and a longer duration of the current depressive episode than in the pivotal study of Popova et al (2019).^5^ The higher treatment resistance may explain the lower absolute rates of remission in both ketamine and control groups compared, for example, with Popova et al (2019). Further, significant efficacy was demonstrated even with assessment of the primary outcome 3 days after the last treatment, rather than the next day as in other key studies ^5^. This is noteworthy given prior findings that efficacy outcomes peak in the day after treatment, then decline over the next few days.^29^ Hence, these results appear compatible with a prior meta-analysis indicating that racemic ketamine showed a larger treatment effect than esketamine,^3^ although clearly uncertainty remains on comparative efficacy and large direct randomised comparisons are required. Onset of the full antidepressant effect of ketamine occurred relatively late in the 4-week period (cohort 2), owing to the gradual dose titration schedule, in which the highest dose (which was required by the majority of participants) could not be attained until the sixth treatment (end of week 3). Overall, an escalating dose titration approach in which dosing is individualised based on clinical response and adverse effects appeared useful, noting the range of final doses across the group of participants, as also found in prior studies.^5,16,17,30^ This approach optimises both efficacy and safety outcomes by individualising the dose required. Future protocols should consider individualised dose titration with more rapid escalation steps so that treatment effects are evident earlier.

### Evaluation of safety

A strength of this study is the comprehensive evaluation of safety, using a prototype of the Ketamine Side Effect Tool (KSET) – a structured instrument that actively examined for immediate, cumulative (between-session) and longer-term side-effects over 4 weeks’ treatment and up to 1 month after the last treatment.^20^ Greater psychotomimetic and cardiovascular acute effects were seen in cohort 2 than in cohort 1, consistent with observations from prior RCTs examining multiple dose levels that effects are dose-related.^16,17^ A few participants (two in the ketamine group, one in the midazolam) reported a sense of wanting the study drug, although this may represent the seeking of relief from depression and anxiety rather than the development of drug dependence. No use of ketamine outside the study protocol was reported. Overall, the treatment showed a good acute, between-session and longer-term safety profile when given within a careful safety monitoring framework. This argues for treatment provision within such a framework, in both clinical and research settings, rather than evidence that acute and cumulative safety monitoring is not required.

The study also evaluated the persistence of benefit with follow-up of all participants 4 weeks after treatment had ended. At 4-week follow-up, remission rates for cohort 2 were 2% *v*. 8% (midazolam *v*. ketamine), with wide confidence intervals and no longer significantly different. This supports other evidence that ongoing treatment is required to maintain antidepressant effects for most participants.^4^ The significant difference in CGI-I and CGI-S scores in cohort 1 at follow-up are difficult to interpret, given the lack of difference on the other outcome measures (remission, response, mean change in MADRS scores) at this same time point, noting that the MADRS provides a more detailed and specific evaluation of depression than the CGI scales.

### Implications for clinical translation

Strengths of the study include the large sample and the use of low-cost, generic ketamine, which is globally available as a result of being on the World Health Organization (WHO) Essential Medicines List as an anaesthetic. Treatment was given by the relatively simple subcutaneous injection method, greatly increasing its clinical translation potential compared with prior parenteral approaches such as a 40 min intravenous infusion, and this treatment approach may be as acceptable to patients as oral or intranasal routes. There was comprehensive evaluation of safety and, to our knowledge, this is the only study using structured assessments of cumulative and between-session effects, as well as acute and overall course effects, and using a tool specifically designed to evaluate adverse effects of ketamine (KSET).

This study provides evidence that racemic ketamine, given by subcutaneous injection at adequate doses, is safe and efficacious in the therapy of treatment-resistant depression over a 4-week treatment course. Benefits attenuate after treatment cessation for most patients, supporting prior evidence that ketamine should be considered as a longer-term treatment. Future research questions include head-to-head comparisons of racemic, R- and S-ketamine, comparisons of routes of administration and testing of treatment strategies to extend benefits and reduce relapse rates.

## Supporting information

Loo et al. supplementary material Loo et al. supplementary material

## Data Availability

De-identified data from this clinical trial may be from the corresponding author on request, subject to approval from the Trial Steering Group and the approving Human Research Ethics Committee.
