# Supplementary material for: Efficacy and safety of a 4-week course of repeated subcutaneous ketamine injections for treatment-resistant depression (KADS study): randomised double-blind active-controlled trial
Source: Br J Psychiatry. 2023 Dec;223(6):533–41. doi: 10.1192/bjp.2023.79 (PMC10727911; doi:10.1192/bjp.2023.79)
Supplement: Loo et al. supplementary material [file S000712502300079Xsup001.docx]

**Supplementary Material - Efficacy and Safety of Repeated Subcutaneous Ketamine Injections for Treatment Resistant Depression – The KADS Study: A Randomised, Double-Blind, Comparator-Controlled Trial.**

**Contents**

[Methods 3](#_Toc127523692)

[Study Sites 3](#_Toc127523693)

[Study Design and Procedures 3](#_Toc127523694)

[Outcomes 4](#_Toc127523695)

[Statistical Analyses 4](#_Toc127523696)

[Results 6](#_Toc127523697)

[CONSORT Diagram 6](#_Toc127523698)

[Figure S1: CONSORT Diagram 6](#_Toc127523699)

[Table of Treatments 7](#_Toc127523700)

[Table S1: Number of doses received by participants in each treatment condition by cohort. 7](#_Toc127523701)

[Sensitivity Analyses of Primary Outcome 7](#_Toc127523702)

[Heterogeneity Analysis of Primary Outcome in Cohort 1 (Fixed-dose) vs Cohort 2 (Flexible-dose) 7](#_Toc127523703)

[Subgroup Analyses 8](#_Toc127523704)

[Figure S2A. Forest plot of subgroup analyses performed on MADRS change scores for Cohort 2 (Flexible-dose) with 90% CIs. 9](#_Toc127523705)

[Figure S2B. Forest plot of subgroup analyses performed on MADRS change scores for Cohort 2 (Flexible-dose) with 95% CIs. 10](#_Toc127523706)

[Analysis of CGI Outcomes 11](#_Toc127523707)

[Figure S3A. CGI Severity – Cohort 1 (Fixed-dose) 12](#_Toc127523708)

[Figure S3B. CGI Severity – Cohort 2 (Flexible-dose) 12](#_Toc127523709)

[Figure S4A. CGI Improvement – Cohort 1 (Fixed-dose) 13](#_Toc127523710)

[Figure S4B. CGI Improvement – Cohort 2 (Flexible-dose) 13](#_Toc127523711)

[Table S2. Analysis of CGI Outcomes as ordinal measures (as described in manuscript) & continuous measures (as per SAP) 14](#_Toc127523712)

[Post RCT Follow-Up at 4-Weeks, 8-Weeks and 6-Months after the last treatment 15](#_Toc127523713)

[Table S3. Primary and Key Secondary Efficacy Outcomes at Post-RCT 4-Week Follow-Up 15](#_Toc127523714)

[Table S4: Mean MADRS, Remission and Response at RCT 8-Week and RCT 6-Month Follow-Ups 16](#_Toc127523715)

[Safety Outcomes 17](#_Toc127523716)

[Figure S5: Mean change in CADSS and BPRS scores across treatment sessions 21](#_Toc127523717)

[Figure S6: Mean changes in systolic and diastolic blood pressure across treatment sessions 22](#_Toc127523718)

[Figure S7: Mean changes in heart rate across treatment sessions 24](#_Toc127523719)

[Table S5: Post-Treatment Adverse Events between treatment sessions. 25](#_Toc127523720)

[Assessment of Blinding 26](#_Toc127523721)

[Table S6: Bang Blinding Index (BBI) for rater and participant, at End RCT, by cohort and treatment group. 27](#_Toc127523722)

[Table S7: Stated reasons for allocation guess, at End RCT, by cohort and treatment group 27](#_Toc127523723)

[References 28](#_Toc127523724)

# Methods

## Study Sites

Recruitment and data collection sites for the study were as follows:

Australia

- Black Dog Institute/UNSW Sydney, NSW
- Royal Prince Alfred Hospital/University of Sydney, NSW
- Monash Alfred Psychiatry Research Centre, VIC
- South Eastern Private Hospital/Neurocentrix, VIC
- Royal Adelaide Hospital/University of Adelaide, SA
- Gold Coast University Hospital, QLD

New Zealand

- University of Otago, Dunedin

At one study site, a pre-screening process was used for multiple trials at that site, with Human Research Ethics Committee approval. Participants at that site were screened using the same eligibility criteria and assessment instruments.

## Study Design and Procedures

A trial statistician computer-generated a permuted-block randomisation. In the randomisation sequence, blocks were a random mixture of size two and four. Treatment allocation was sequential.

Each treatment was given at a trial centre with monitoring of vital signs and psychotomimetic effects for 4 hours after dosing at the first treatment and 2 hours after dosing at subsequent treatments. Secondary outcomes were assessed at RCT baseline and at end. End of RCT assessments were done 3-4 days after the last treatment, with follow up assessments 4 weeks later, followed by a 4-week optional open label treatment phase (to be reported elsewhere). Those who did not enter open label treatment were followed up at 8 weeks and 6 months after RCT end. See Consort diagram (Figure S1).

To facilitate blinding, different blinded assessors (“raters”) rated mood, anxiety, and suicidality outcomes, and assessed safety effects of ketamine at each site. Raters were excluded from observation of treatment sessions. Participants were instructed at each rating interview not to mention any treatment-related experience to the raters.

## Outcomes

Safety assessments at each treatment session included: (i) a pre-treatment Ketamine Side Effect Tool (KSET) (1), checklist assessing for cumulative or emerging (between session) effects of repeated ketamine treatments, including urinary symptoms and ketamine craving; (ii) suicidal ideation scale of the Columbia Suicide Severity Rating Scale (2), assessed pre-treatment; (iii) blood pressure and heart rate, measured pre-treatment and at 15 minutes, 1 and 2 hours (and 4 hours at first session) after treatment; (iv) Clinician Administered Dissociative States Scale (3), positive items of the Brief Psychiatric Rating Scale (4), item 1 (elevated mood) of the Young Mania Rating Scale (5), and KSET acute checklist, at 1 hour (rating peak effects over the last 60 minutes) and 2 hours (checking for resolution, i.e., rating of effects as at 120 minutes) after treatment; (v) KSET checklist assessing fitness for discharge, including orientation and return of vital signs to ≤ 120% of pre-treatment levels.

At RCT baseline, end RCT, and 4-week follow up, liver function tests, bladder function (Bladder Pain Interstitial Cystitis Symptom Score)(6), and KSET checklist (including questions on ketamine craving or use outside protocol) were assessed, as well as a battery of cognitive tests assessing attention, psychomotor function, working memory, frontal function, verbal and visual memory (see trial protocol for details).

Full details of all outcomes are available in the trial protocol and statistical analysis plan. Changes to trial outcomes after the commencement of the trial are summarised in the Clinical Trial Protocol Revision History.

## Statistical Analyses

The initial recruitment goal was n=100 per arm, based on power of at least 90% for a difference in remission proportions of 30% versus 10% (requiring n=82 per arm), plus an inflation to allow for participant loss. Following the protocol change the goal for Cohort 2 was n=62 per arm based on power of 80% (with no inflation, reflecting the minimal loss observed in Cohort 1).

Analyses initially fitted linear mixed effects models (LME) to allow for random effects (if numerically feasible). Generalised LMEs for binary outcomes (such as remission) frequently had numerical issues associated with low numbers of events (such as cells with zero events, complete separability) in which case estimates were obtained from a logistic regression with Firth’s penalized likelihood (PLR).

Analyses for outcomes (e.g., remission) which were derived from MADRS scores at individual time points used multiple-imputed datasets of the MADRS. Outcomes (e.g., change from baseline) estimated in mixed effect models for repeated measures (MERM, which use all observed data from all occasions) did not use imputed data. Covariance structures for repeats were selected using information criteria.

For multiple imputation (MI) 100 imputed datasets were created using fully conditional specification (FCS) with observed data in multivariate format.

MI analyses report estimates and CIs as output by the software. Otherwise, we report standard estimates and Wald-type confidence intervals (CI), except for PLR where profile-likelihood estimates and intervals – which are likely to be more accurate – are reported (if available). All CIs are 95%, except for subgroup analyses (see Subgroup Analysis section below). No adjustments for multiple inference were made.

# Results

## CONSORT Diagram

### Figure S1: CONSORT Diagram


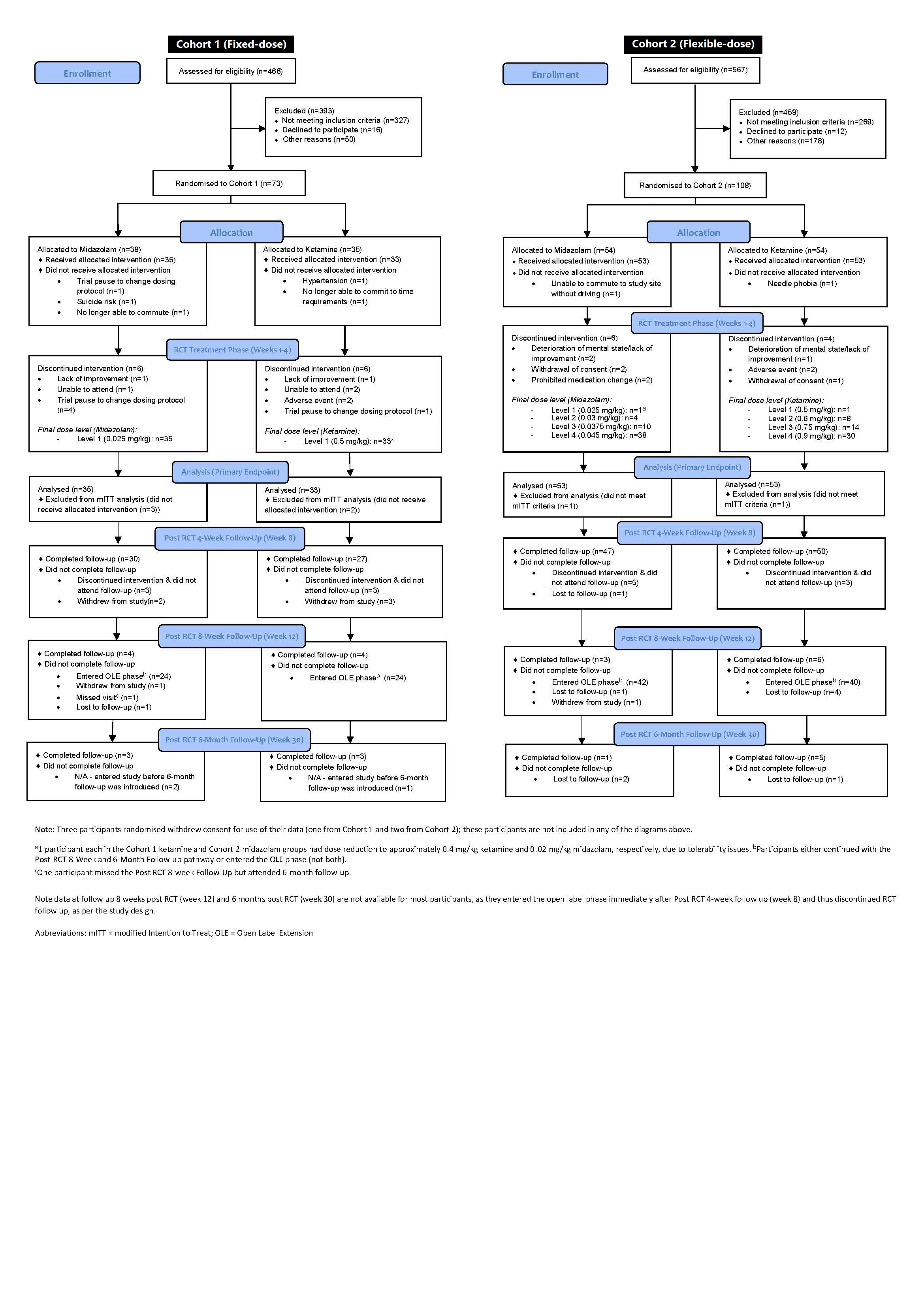


## Table of Treatments

### Table S1: Number of doses received by participants in each treatment condition by cohort.

|  | **Cohort 1 (Fixed-dose)** | | | | **Cohort 2 (Flexible-dose)** | | | |
| --- | --- | --- | --- | --- | --- | --- | --- | --- |
|  | **Midazolam** | | **Ketamine** | | **Midazolam** | | **Ketamine** | |
|  | n | (%) | n | (%) | n | (%) | n | (%) |
| At least 1 dose | 35 | (100.0) | 33 | (100.0) | 53 | (100.0) | 53 | (100.0) |
| At least 2 doses | 35 | (100.0) | 31 | (93.9) | 53 | (100.0) | 53 | (100.0) |
| At least 3 doses | 34 | (97.1) | 29 | (87.9) | 51 | (96.2) | 52 | (98.1) |
| At least 4 doses | 32 | (91.4) | 29 | (87.9) | 50 | (94.3) | 52 | (98.1) |
| At least 5 doses | 30 | (85.7) | 29 | (87.9) | 50 | (94.3) | 50 | (94.3) |
| At least 6 doses | 30 | (85.7) | 28 | (84.8) | 50 | (94.3) | 50 | (94.3) |
| At least 7 doses | 26 | (74.3) | 27 | (81.8) | 45 | (84.9) | 49 | (92.5) |
| All 8 doses | 19 | (54.3) | 26 | (78.8) | 40 | (75.5) | 43 | (81.1) |

## Sensitivity Analyses of Primary Outcome

Sensitivity analyses using a “tipping point” approach examined the assumptions for missing data. With only 4/53 missing values under midazolam and 2/53 under ketamine, we based a sensitivity analysis for Cohort 2 on analysing all possible assignments of remission or non-remission to those 6 participants. Only two of the 64 datasets had a profile-likelihood CI that included 1. In one case (the worst-case) all midazolam participants were set to remission and all ketamine participants set to non-remission (OR = 2.74, CI = 0.86 to 9.85). The other case differed by setting one specific ketamine participant to remission (OR = 3.07, CI = 1.00 to 10.79). Thus, only these two extreme-case scenarios represent “tipping-points”.

## Heterogeneity Analysis of Primary Outcome in Cohort 1 (Fixed-dose) vs Cohort 2 (Flexible-dose)

Analysis in Observed data.

A logistic regression was fitted in PROC LOGISTIC with cohort, site, treatment arm, baseline MADRS, and treatment arm × cohort as fixed effects. The estimate indicated that the odds favouring Ketamine in Cohort 2 were 12.88 times the odds favouring Ketamine in Cohort 1 (95% CI = 1.10 to 151.33, p=0.042).

Analysis in Imputed data.

The model was also fitted in the imputed datasets. The result was similar: the estimate indicated that the odds favouring Ketamine in Cohort 2 were 13.0 times the odds favouring Ketamine in Cohort 1 (95% CI 1.10 to 152.54, p=0.042).

## Subgroup Analyses

A series of subgroup analyses was carried out in Cohort 2 only, using change in MADRS as the outcome. (The planned use of remission was not feasible with only a single remission for midazolam in Cohort 2.) Subgroup analyses are typically underpowered hence we set α = 0.10, with no adjustment for multiple inference, as recommended to better capture signals in the data (7). Of the 17 subgroups analysed, 3 interactions were significant: the superiority of ketamine was greater (i) in those with high baseline anxiety; (ii) in those currently on antipsychotics; and (iii) in those who had failed more antidepressants in their lifetime. Figure S2A shows a forest plot of the within-subgroup levels differences between ketamine and midazolam expressed as estimates plus their 90% CIs. As some readers might prefer a less liberal analysis, Figure S2B shows 95% CIs. We further note that if p values were adjusted for the false discovery rate, then only interaction (ii) would remain significant (at α = 0.10) and none would do so at α = 0.05.

### **Figure S2A. Forest plot of subgroup analyses performed on MADRS change scores for Cohort 2 (Flexible-dose) with 90% CIs.**


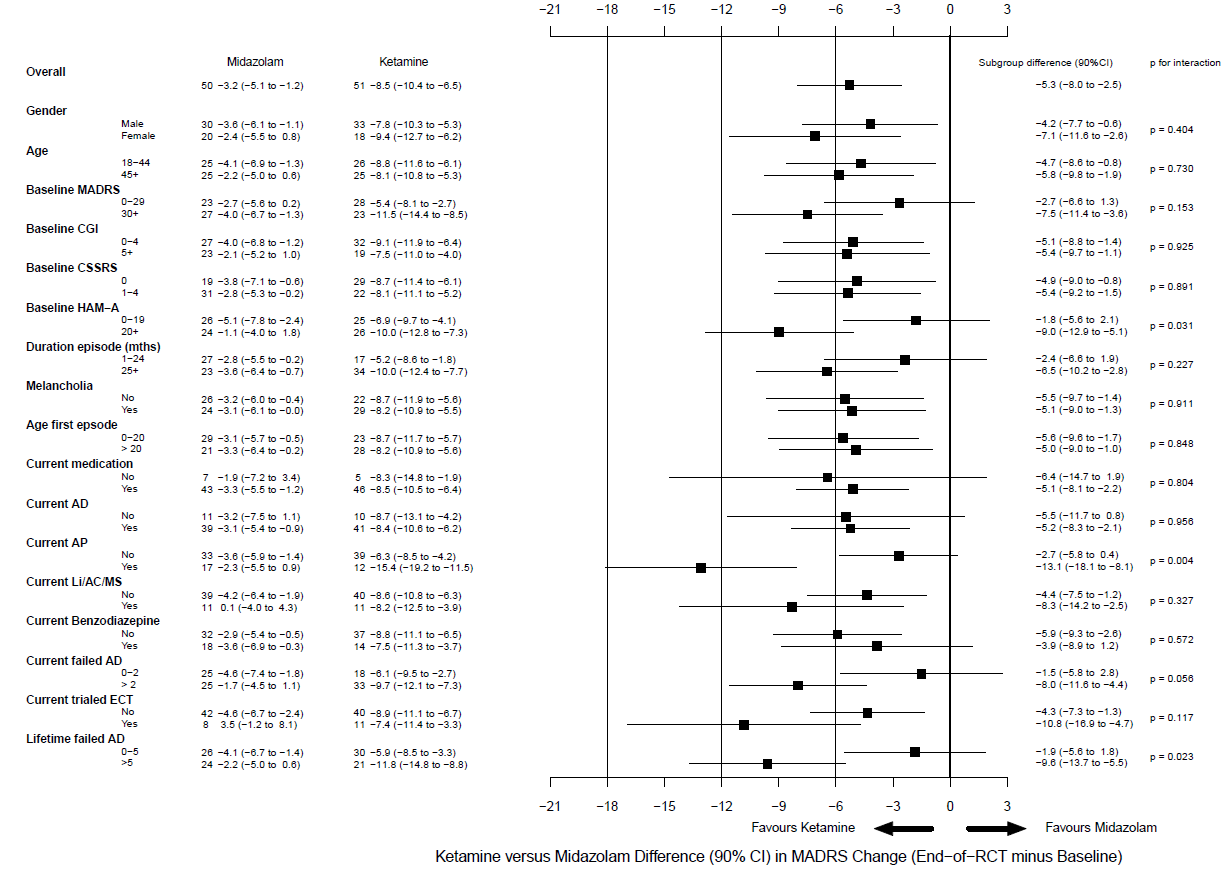


Abbreviations: AD = antidepressant; AP = antipsychotic; CGI = Clinical Global Impression; CSSRS = Columbia Suicide Severity Rating Scale; ECT = Electroconvulsive Therapy; HAM-A = Hamilton Anxiety Rating; Li/AC/MS = Lithium/Anticonvulsant/Mood Stabiliser; MADRS = Montgomery- Åsberg Depression Rating Scale.

### Figure S2B. Forest plot of subgroup analyses performed on MADRS change scores for Cohort 2 (Flexible-dose) with 95% CIs.


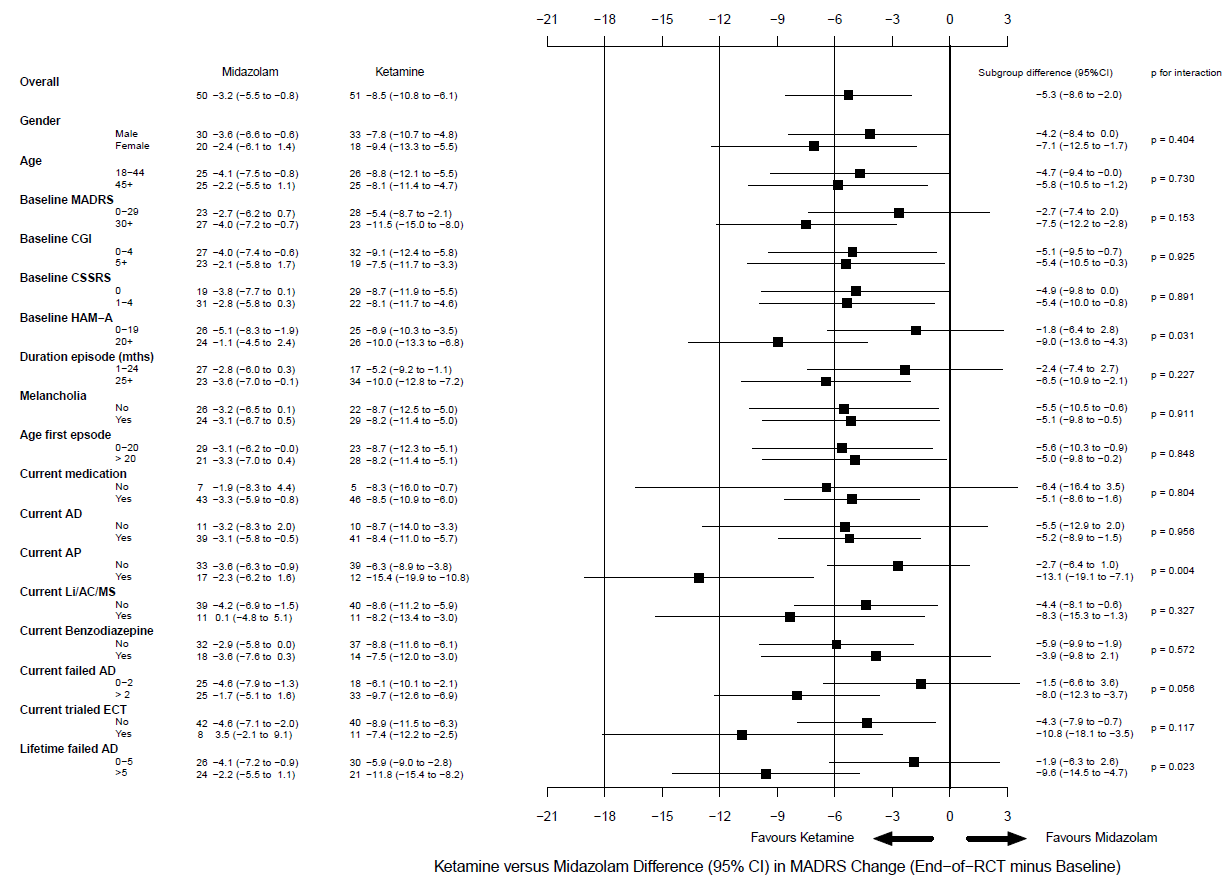


Abbreviations: AD = antidepressant; AP = antipsychotic; CGI = Clinical Global Impression; CSSRS = Columbia Suicide Severity Rating Scale; ECT = Electroconvulsive Therapy; HAM-A = Hamilton Anxiety Rating; Li/AC/MS = Lithium/Anticonvulsant/Mood Stabiliser; MADRS = Montgomery- Åsberg Depression Rating Scale.

## Analysis of CGI Outcomes

While the SAP indicated that CGI Severity would be analysed as a continuous measure of change from baseline, examination of the observed data, and reconsideration of the basis underlying the CGI indicated that it would be better analysed as ordinal categories. Additional analyses of CGI scores as continuous measures (as per the SAP) are also provided below.

There were two observations for CGI Severity with a score of 7 (i.e., highest severity score on the 1-7 point scale), one in Cohort 1 (Midazolam) at 4 weeks; and one in Cohort 2 (Midazolam) at end of RCT. These were collapsed into a score of 6 (in both cases it would favour Midazolam).

Analysis used PROC GLIMMIX to fit an ordinal regression (multinomial distribution with a CLOGIT link). Adjusted for baseline CGI Severity (coded as categories) and SITE.

Models with random SITE in most cases produced a numerical issue (nonpositive-definite G matrix; zero estimates for all SITEs). We have therefore reported models with fixed effects for SITE.

Modelling probability of *lower* response. TRT effect > 0 (OR > 1) favours Ketamine.

In the outcomes below, **bolded** values represent significant differences between the treatment groups.

### Figure S3A. CGI Severity – Cohort 1 (Fixed-dose)

**
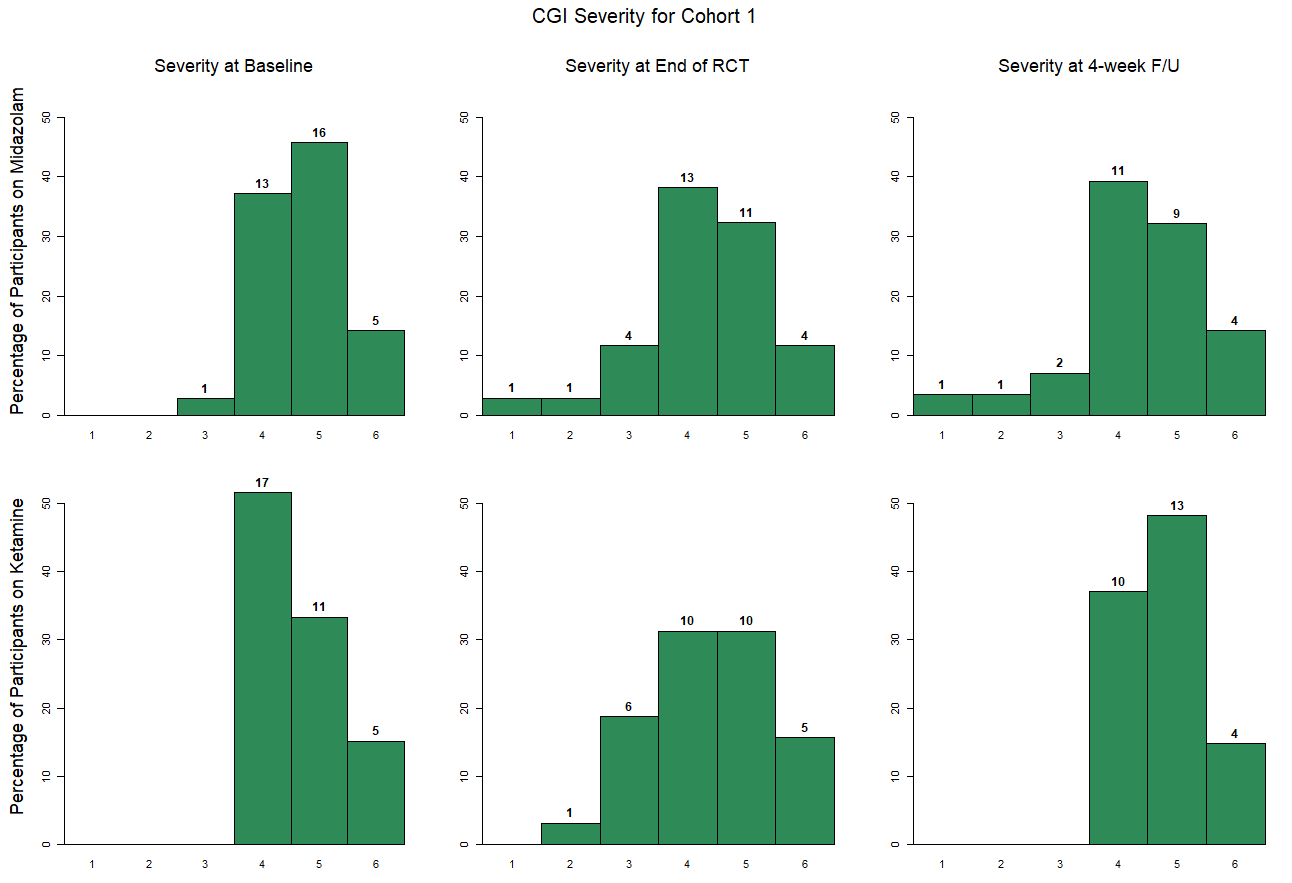
**

### Figure S3B. CGI Severity – Cohort 2 (Flexible-dose)

**
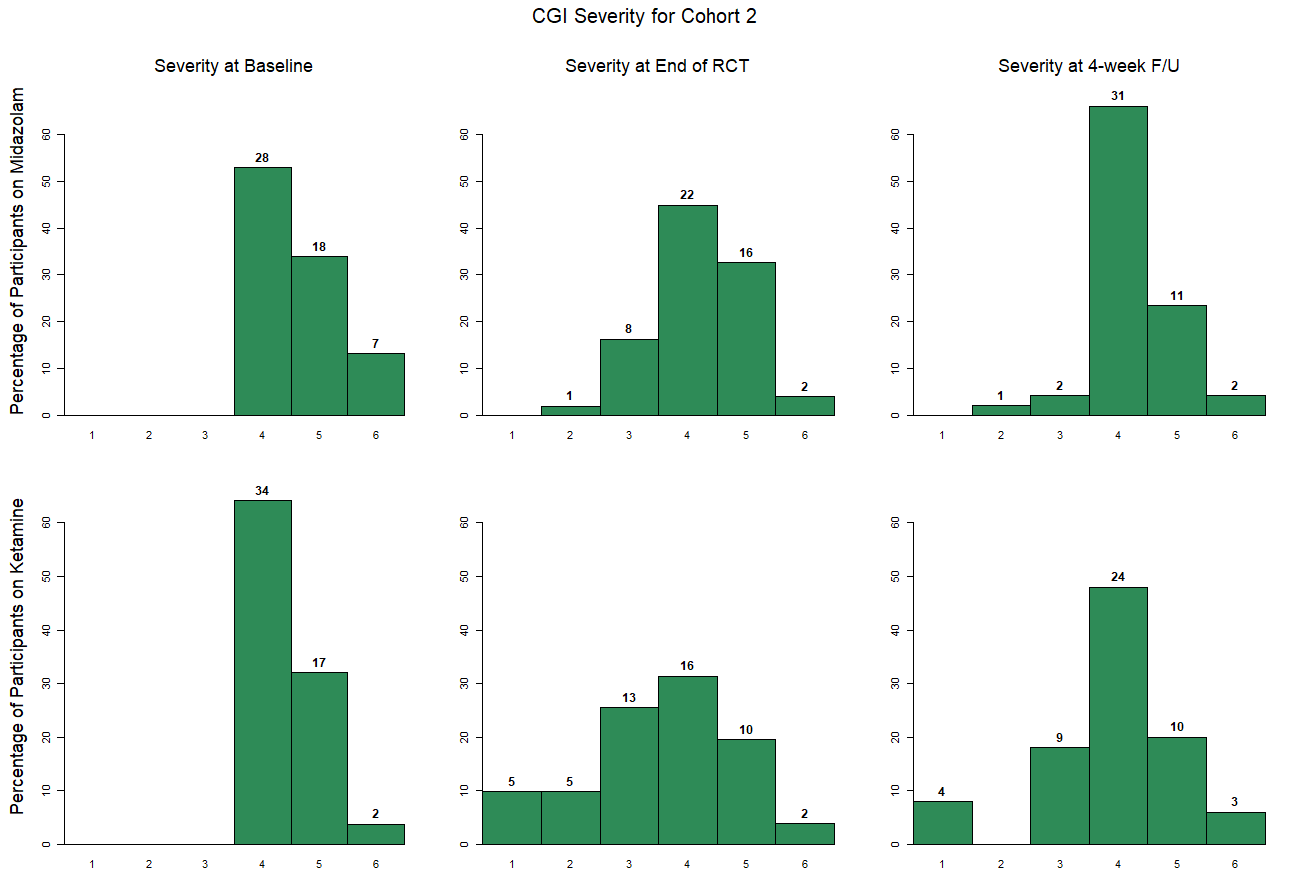
**

### Figure S4A. CGI Improvement – Cohort 1 (Fixed-dose)


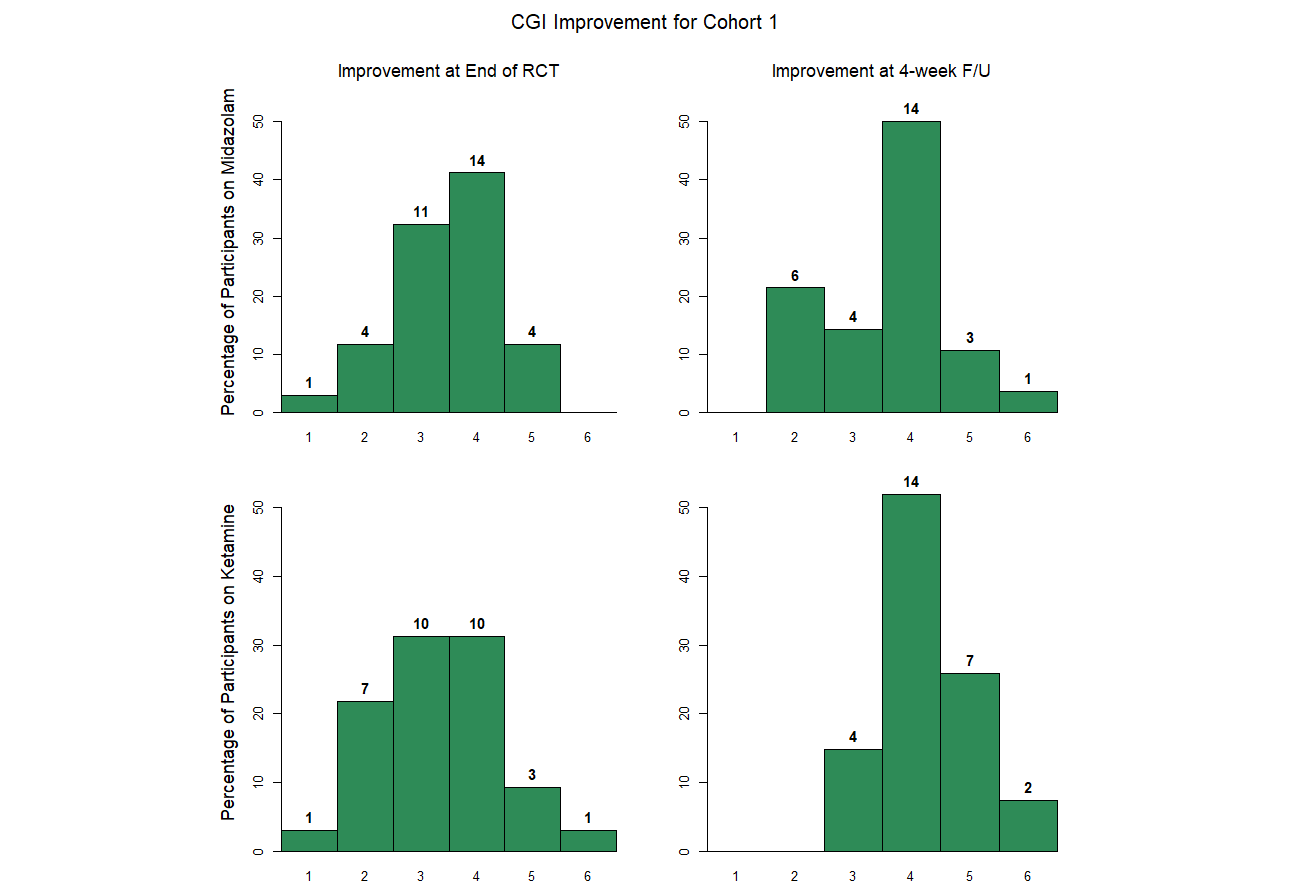


### Figure S4B. CGI Improvement – Cohort 2 (Flexible-dose)

**
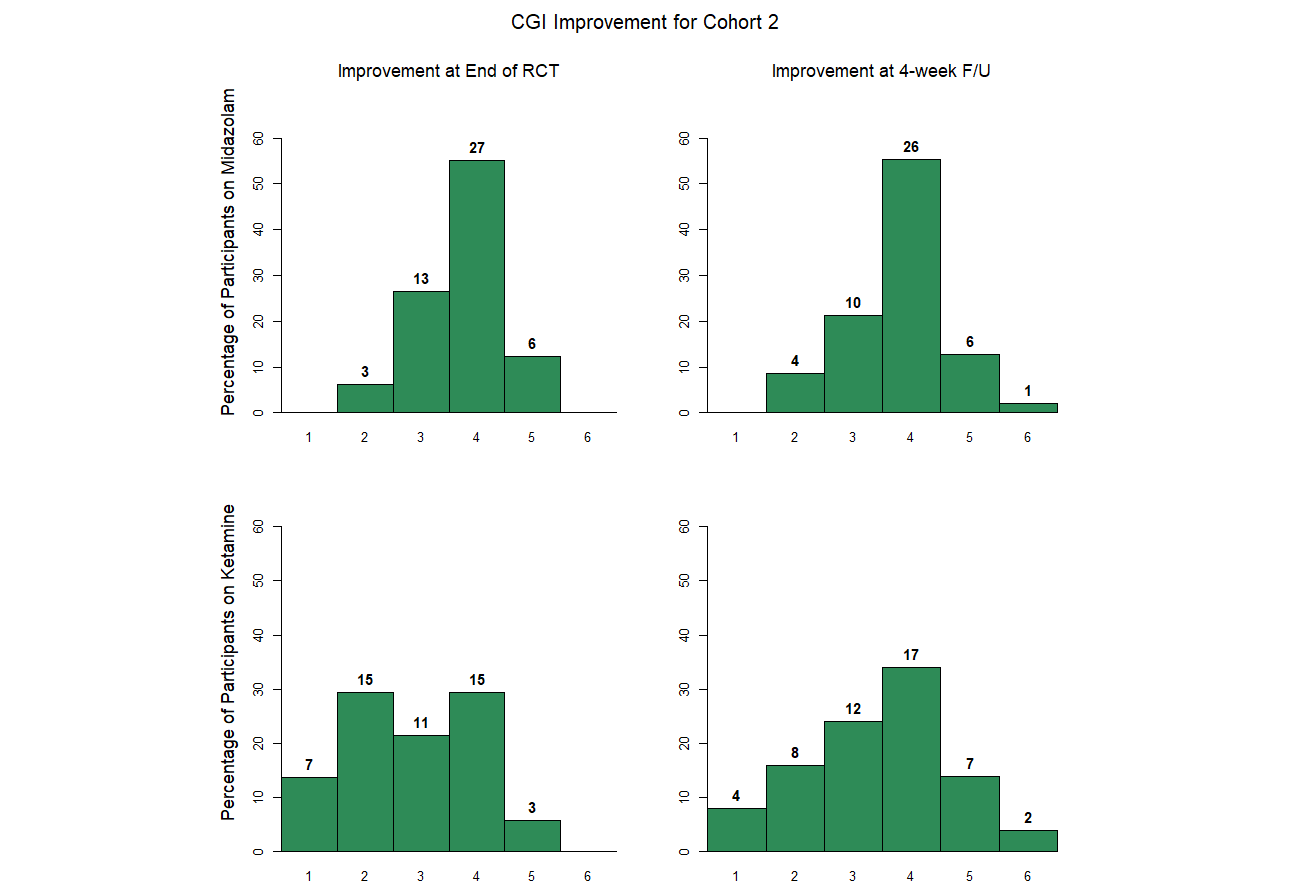
**

### Table S2. Analysis of CGI Outcomes as ordinal measures (as described in manuscript) & continuous measures (as per SAP)

|  | **Cohort 1 (Fixed-dose)** | | | | | | | **Cohort 2 (Flexible-dose)** | | | | | | |
| --- | --- | --- | --- | --- | --- | --- | --- | --- | --- | --- | --- | --- | --- | --- |
|  | **Midazolam** | | **Ketamine** | | **Treatment effect estimate, 95% CI and p-value** | | | **Midazolam** | | **Ketamine** | | **Treatment effect estimate, 95% CI and p-value** | | |
| N (mITT/End RCT/ Post RCT 4-wk F/U)^a^ | 35/34/28 | | 33/32/27 | |  | | | 53/49/47 | | 53/51/50 | |  | | |
|  |  | |  | | **Ketamine/Midazolam** | | |  | |  | | **Ketamine/Midazolam** | | |
|  | **N** | **(%)** | **N** | **(%)** | **aOR** | **95%CI** | **p** | **N** | **(%)** | **N** | **(%)** | **aOR** | **95%CI** | **p** |
| CGI-S^c^ at End RCT |  | |  | |  |  |  |  | |  | |  |  |  |
| ≤ 3(“Mild”) | 6 | (17.6) | 7 | (21.9) | 0.63 | 0.23 to 1.67 | 0.34 | 9 | (18.4) | 23 | (45.1) | **2.65** | **1.20 to 5.84** | **0.02** |
| ≤ 4 (“Moderate”) | 19 | (55.9) | 17 | (53.1) | NA | NA | NA | 31 | (63.3) | 39 | (76.5) | NA | NA | NA |
| CGI-S^c^ at Post RCT 4-wk F/U |  | |  | |  |  |  |  | |  | |  |  |  |
| ≤ 3(“Mild”) | 4 | (14.3) | 0 | (0.0) | **0.25** | **0.07 to 0.89** | **0.03** | 3 | (6.4) | 13 | (26.0) | 1.35 | 0.59 to 3.13 | 0.47 |
| ≤ 4 (“Moderate”) | 15 | (53.6) | 10 | (37.0) | NA | NA | NA | 34 | (72.3) | 37 | (74.0) | NA | NA | NA |
|  |  |  |  |  |  |  |  |  |  |  |  |  |  |  |
| CGI-I^c^ at End RCT |  | |  | |  |  |  |  | |  | |  |  |  |
| ≤ 2 (“Much”) | 5 | (14.7) | 8 | (25.0) | 1.18 | 0.45 to 3.07 | 0.73 | 3 | (6.1) | 22 | (43.1) | **5.74** | **2.51 to 13.14** | **<0.001** |
| ≤ 3 (“Minimal”) | 16 | (47.1) | 18 | (56.2) | NA | NA | NA | 16 | (32.7) | 33 | (64.7) | NA | NA | NA |
| ≤ 4 (“No change”) | 30 | (88.2) | 28 | (87.5) | NA | NA | NA | 43 | (87.8) | 48 | (94.1) | NA | NA | NA |
| CGI-I^c^ at Post RCT 4-wk F/U |  | |  | |  |  |  |  | |  | |  |  |  |
| ≤ 2 (“Much”) | 6 | (21.4) | 0 | (0.0) | **0.21** | **0.06 to 0.68** | **0.010** | 4 | (8.5) | 12 | (24.0) | 1.89 | 0.88 to 4.08 | 0.10 |
| ≤ 3 (“Minimal”) | 10 | (35.7) | 4 | (14.8) | NA | NA | NA | 14 | (29.8) | 24 | (48.0) | NA | NA | NA |
| ≤ 4 (“No change”) | 24 | (85.7) | 18 | (66.7) | NA | NA | NA | 40 | (85.1) | 41 | (82.0) | NA | NA | NA |
|  |  |  |  |  |  |  |  |  |  |  |  |  |  |  |
|  |  |  |  |  | **Ketamine - Midazolam** | | |  |  |  |  | **Ketamine - Midazolam** | | |
|  | **M** | **SD** | **M** | **SD** | **Δ** | **95%CI** | **p** | **M** | **SD** | **M** | **SD** | **Δ** | **95%CI** | **p** |
| Change in CGI-S from baseline |  |  |  |  |  |  |  |  |  |  |  |  |  |  |
| End RCT | 0.44 | (0.89) | 0.28 | (0.81) | −0.16 | −0.60 to 0.28 | 0.48 | 0.35 | (0.86) | 0.88 | (1.29) | **0.60** | **0.19 to 1.02** | **0.005** |
| Post-RCT 4-wk F/U | 0.29 | (0.90) | −0.15 | (0.53) | **−0.41** | **−0.82 to −0.001** | **0.049** | 0.32 | (0.66) | 0.50 | (1.13) | 0.21 | −0.16 to 0.58 | 0.26 |
|  |  |  |  |  |  |  |  |  |  |  |  |  |  |  |
| CGI-I |  |  |  |  |  |  |  |  |  |  |  |  |  |  |
| End RCT | 3.47 | (0.96) | 3.31 | (1.12) | −0.12 | −0.60 to 0.36 | 0.62 | 3.73 | (0.76) | 2.84 | (1.17) | **−0.92** | **−1.32 to −0.520** | **< 0.0001** |
| Post-RCT 4-wk F/U | 3.61 | (1.07) | 4.26 | (0.81) | **0.65** | **0.15 to 1.16** | **0.010** | 3.79 | (0.86) | 3.42 | (1.26) | −0.40 | −0.85 to 0.05 | 0.084 |

^a^For End RCT and 4-wk F/U, N reflects the participants with a CGI score available at the respective time points. ^b^ Adjusted OR (aOR) > 1 favour Ketamine. ^c^ Data reported for CGI-S and CGI-I categories reflect the cut-off points used in the analysis; percentages do not add to 100%.

## Post RCT Follow-Up at 4-Weeks, 8-Weeks and 6-Months after the last treatment

### Table S3. Primary and Key Secondary Efficacy Outcomes at Post-RCT 4-Week Follow-Up

|  | **Cohort 1 (Fixed-dose)** | | | | | | | **Cohort 2 (Flexible-dose)** | | | | | | |
| --- | --- | --- | --- | --- | --- | --- | --- | --- | --- | --- | --- | --- | --- | --- |
|  | **Midazolam** | | **Ketamine** | | **Treatment effect estimate, 95% CI and p-value** | | | **Midazolam** | | **Ketamine** | | **Treatment effect estimate, 95% CI and p-value** | | |
| N | 29 | | 27 | |  |  |  | 47 | | 50 | |  |  |  |
| MADRS total score  (Mean (SD)) | 28.2 (10.0) | | 30.4 (4.9) | |  |  |  | 27.7 (6.7) | | 25.8 (9.0) | |  |  |  |
|  |  | |  | |  |  |  |  | |  | |  |  |  |
|  |  | |  | | **Ketamine/Midazolam** | | |  | |  | | **Ketamine/Midazolam** | | |
| Remission MADRS ≤ 10 | **N** | **(%)** | **N** | **(%)** | **aOR^b^** | **95% CI** | **p** | **N** | **(%)** | **N** | **(%)** | **aOR^b^** | **95% CI** | **p** |
| 4-wk F/U | 2 | (6.9) | 0 | (0.0) | 0.47 | 0.04 to 5.49 | 0.55 | 1 | (2.1) | 4 | (8.0) | 2.02 | 0.40 to 10.28 | 0.40 |
| Remission MADRS ≤ 12 |  | |  | |  |  |  |  | |  | |  |  |  |
| 4-wk F/U | 2 | (6.9) | 0 | (0.0) | 0.47 | 0.04 to 5.49 | 0.55 | 1 | (2.1) | 5 | (10.0) | 3.02 | 0.60 to 15.15 | 0.18 |
| Response (MADRS change ≥ 50%) |  | |  | |  |  |  |  | |  | |  |  |  |
| 4-wk F/U | 2 | (6.9) | 0 | (0.0) | 0.44 | 0.04 to 5.58 | 0.53 | 1 | (2.1) | 5 | (10.0) | 2.95 | 0.59 to 14.76 | 0.19 |
|  |  | |  | | **Ketamine − Midazolam** | | |  | |  | | **Ketamine – Midazolam** | | |
| Change in MADRS (from BL) | **M** | **(SD)** | **M** | **(SD)** | **Δ** | **95%CI** | **p** | **M** | **(SD)** | **M** | **(SD)** | **Δ** | **95%CI** | **p** |
| 4-wk F/U | −2.10 | (8.33) | 0.33 | (4.22) | 2.32 | −1.05 to 5.68 | 0.17 | −2.45 | (5.54) | −2.90 | (8.50) | −0.79 | −3.65 to 2.08 | 0.59 |

^a^N reflects the participants with a MADRS score available at the Post-RCT 4-Week Follow-Up time point. ^b^ Adjusted OR (aOR) > 1 favour Ketamine.

### Table S4: Mean MADRS, Remission and Response at RCT 8-Week and RCT 6-Month Follow-Ups

|  | **Cohort 1** **(Fixed-dose)** | | | | **Cohort 2** **(Flexible-dose)** | | | |
| --- | --- | --- | --- | --- | --- | --- | --- | --- |
|  | **Midazolam** | | **Ketamine** | | **Midazolam** | | **Ketamine** | |
| N |  | |  | |  | |  | |
| 8-wk F/U | 4 | | 4 | | 3 | | 6 | |
| 6-month F/U | 3 | | 3 | | 1 | | 5 | |
|  |  | |  | |  | |  | |
| MADRS total | **Mean** | **(SD)** | **Mean** | **(SD)** | **Mean** | **(SD)** | **Mean** | **(SD)** |
| 8-wk F/U | 36.5 | (11.8) | 27.5 | (1.9) | 23.3 | (16.8) | 13.7 | (12.3) |
| 6-month F/U | 36.3 | (13.6) | 27.0 | (3.0) | 21.0^a^ | - | 13.6 | (9.7) |
|  |  | |  | |  | |  | |
| Remission MADRS ≤ 10 | **n** | **(%)** | **n** | **(%)** | **n** | **(%)** | **n** | **(%)** |
| 8-wk F/U | 0 | (0.0) | 0 | (0.0) | 1 | (33.3) | 4 | (66.7) |
| 6-month F/U | 0 | (0.0) | 0 | (0.0) | 0 | (0.0) | 2 | (40.0) |
|  |  | |  | |  | |  | |
| Response (MADRS change ≥ 50%) | **n** | **(%)** | **n** | **(%)** | **n** | **(%)** | **n** | **(%)** |
| 8-wk F/U | 0 | (0.0) | 0 | (0.0) | 1 | (33.3) | 4 | (66.7) |
| 6-month F/U | 0 | (0.0) | 0 | (0.0) | 0 | (0.0) | 3 | (60.0) |

^a^SD not reported as n=1 for this cell

Note that follow up data at 8 weeks and 6 months after the RCT are not available for most participants, as they entered the open label phase and thus discontinued RCT follow up, as per the study design.

## Safety Outcomes

Medically Significant Adverse Events and Serious Adverse Events (SAEs)

There were no deaths throughout the study. Two participants experienced medically significant events which were judged to be related to the study drug, both in the Cohort 2 ketamine group:

- Acute major dissociative episode at RCT treatment 6, in the context of an incorrect dose up-titration* to 0.9 mg/kg ketamine. The CADSS score peaked at 66 at 1 hour, returning to 0 at 2 hours; BPRS positive subscale was 24 at 1 hour, 5 at 2 hours. BP increased from 123/86 pre treatment to 179/94 at 30 minutes. HR increased from 75 pre treatment to 136 at 30 minutes post treatment. All symptoms resolved, mostly within an hour and entirely by 2 hours, without need for any medical intervention.
- Auditory hallucination at RCT treatment 8 wherein the participant heard a voice saying the researcher was trying to poison the participant, which resolved within 1 hour of treatment. The participant later stated she did not feel compelled to do anything as a result. This was in the context of an incorrect dose up-titration to 0.9 mg/kg ketamine. No medical treatment was required. *Dose titration protocol errors (e.g., dose increase despite participant meeting MADRS response criteria; titration occurring at incorrect treatment session) were recorded for 2/392 midazolam treatment sessions (0.5%, n=2) and 6/401 ketamine treatment sessions (1.5%, n=5) in RCT Cohort 2.

Two suicide attempts were reported (1 participant each in the midazolam groups of Cohorts 1 and 2), but both were judged to be unlikely related to the study drug:

- Cohort 1 midazolam: Suicide attempt by overdose in a participant with chronic suicidal ideation and a history of self-harm, resulting in hospitalisation. The participant had been withdrawn from the study intervention due to lack of improvement and this event occurred 10 days after their last dose.
- Cohort 2 midazolam: Suicide attempt by self-injury after the participant felt angry following an argument. The event occurred on Day 20 post-baseline, 3 days after their last dose, and resulted in hospital admission.

There was 1 SAE which was judged as being unlikely related to the study drug.

- Cohort 1 midazolam (n=1): Voluntary hospitalisation for respite stay on post-baseline Day 25 for increased suicidal ideation, assessed by the investigator as being likely related to social stressors.

All other SAEs were hospitalisations judged to be unrelated to the study drug.

- Hospitalisation for mood deterioration (1 participant in Cohort 2 midazolam)
- Wrist injury caused by a fall sustained before entering the study (1 participant in Cohort 2 midazolam)

AE-related treatment withdrawals

Four participants withdrew from study treatment as a result of adverse events; all were in the ketamine condition, with 2 each in Cohorts 1 and 2.

- Cohort 1 ketamine: Pruritic skin rash which emerged approximately 3-4 hours after receiving first dose of the study drug at treatment 1. Judged to be a potential allergic reaction to the study drug and withdrawn from treatment by the study doctor.
- Cohort 1 ketamine: Exacerbation in anxiety post-injection at treatment 2 which lasted for 3 hours. Participant chose to withdraw from further treatment.
- Cohort 2 ketamine: Severe tension headaches emerging 12-36 hours post-injection in context of history of headaches. Participant chose to withdraw due to tolerability.
- Cohort 2 ketamine: Confusion and depressed state in the 1-2 days following treatment. Participant chose to withdraw.

Dissociation and Psychotomimetic Symptoms

Figure S5 shows mean changes in CADSS and BPRS scores at 60- and 120-minutes post injection for the ketamine and midazolam groups at each treatment session. Ratings at 60 minutes post-injection reflect symptoms experienced over the past hour; ratings at 120 minutes post injection reflect current symptoms at the time. CADSS and BPRS scores were higher in the hour after treatment, and more evident in Cohort 2 than Cohort 1.

Blood Pressure and Heart Rate

Figure S6 shows mean changes in systolic and diastolic blood pressure across treatment sessions. Blood pressure measurements were taken pre-injection, and at 15 minutes, 60 minutes, and 120 minutes post injection at each treatment session. Blood pressure increased after ketamine treatment, more evident in Cohort 2 than Cohort 1 (see Figure S6). Clinically relevant blood pressure increases (i.e., systolic blood pressure ≥ 180 mmHg and increase ≥ 20mmHg, diastolic blood pressure ≥ 105 mmHg and increase of ≥ 15 mmHg)(8) were more evident in the ketamine group for both cohorts (Cohort 1: midazolam 1/35 (2.9%), ketamine 2/33 (6.1%); Cohort 2: midazolam 0/53 (0.0%), ketamine 3/53 (5.7%)).

Figure S7 shows mean changes in heart rate across treatment sessions. Measurements were taken pre-injection, and at 15 minutes, 60 minutes and 120 minutes post injection at each treatment session. There were no major differences between treatment arms or the two cohorts.

Fitness for Discharge

A few participants stayed at the centre for up to an additional 60 minutes to fully meet discharge criteria on at least one occasion (Cohort 1: midazolam 3/35 (8.6%), ketamine 2/33 (6.1%); Cohort 2: midazolam 6/53 (11.3%), ketamine 9/53 (17.0%)).

K-SET Section A

Table S5 shows results from the K-SET Section A, which reflects new onset of, or exacerbation of, symptoms observed since the participant’s last ketamine or midazolam treatment. The incidence of participants experiencing each adverse event was compared between groups using a Fisher’s exact test. The only symptoms that were evident between sessions were higher scores in the ketamine group for headache and increased energy, in Cohort 2.

Liver Function & Urological Symptoms

No clinically significant changes in liver function test results were evident after 4 weeks of treatment.

The number of participants reporting at least one new urological symptom (pain, increased frequency, burning, change in urine colour, reduced force of urinary stream) at RCT End was similar between groups for Cohort 1 (midazolam = 4/35 (11.4%), ketamine = 4/33 (12.1%)) but higher in the ketamine group for Cohort 2 (midazolam = 1/53 (1.9%), ketamine = 3/53 (5.7%)). There was little change in BPIC-SS scores after 4 weeks of treatment for both cohorts, with a mean change of -0.29 (SD=2.76) and 0.19 (SD=2.81) for the Cohort 1 midazolam and ketamine groups (respectively), and a mean change of 0.04 (SD=1.89) and -0.75 (SD=3.17) for the Cohort 2 midazolam and ketamine groups. No participant had a clinically significant BPIC-SS score of ≥19 (6) at RCT End.

Craving and Recreational Use

Three participants reported craving for the study drug during the 4-week treatment period, all in cohort 2 (ketamine n=2, midazolam n=1). No participants reported using ketamine outside of supervised medical treatment or research up to the RCT 4-week follow-up in either cohort.

### **Figure S5: Mean change in CADSS and BPRS scores across treatment sessions**

Measurements were taken at 60 and 120 minutes post injection.


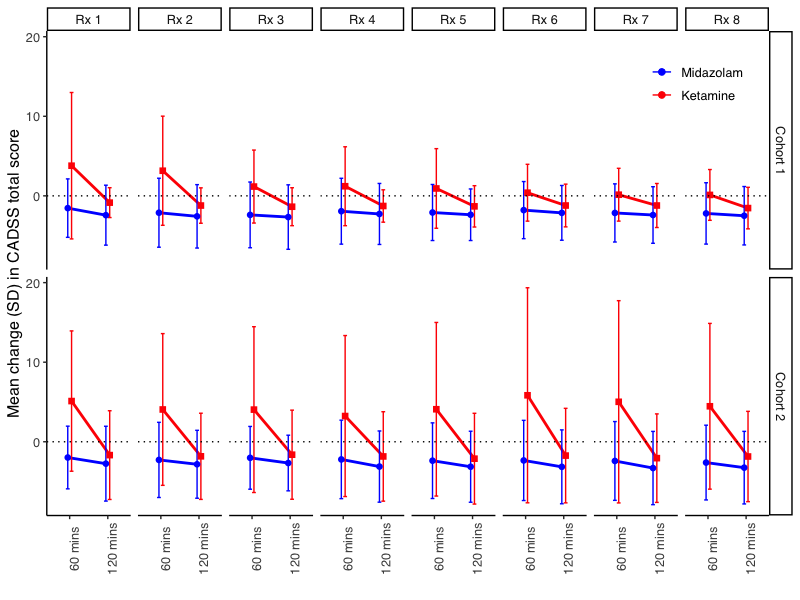


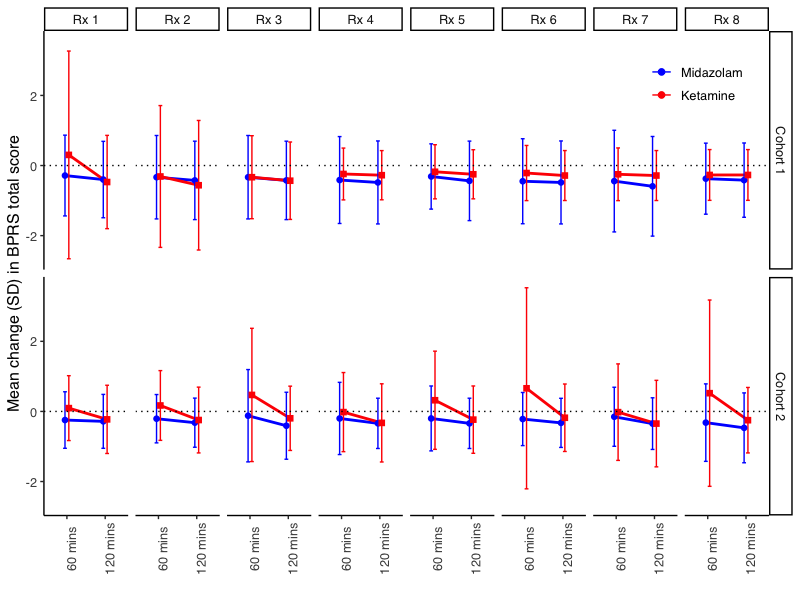


### **Figure S6: Mean changes in systolic and diastolic blood pressure across treatment sessions**

Blood pressure measurements were taken pre-injection, and at 15 minutes, 60 minutes and 120 minutes post injection at each treatment session.


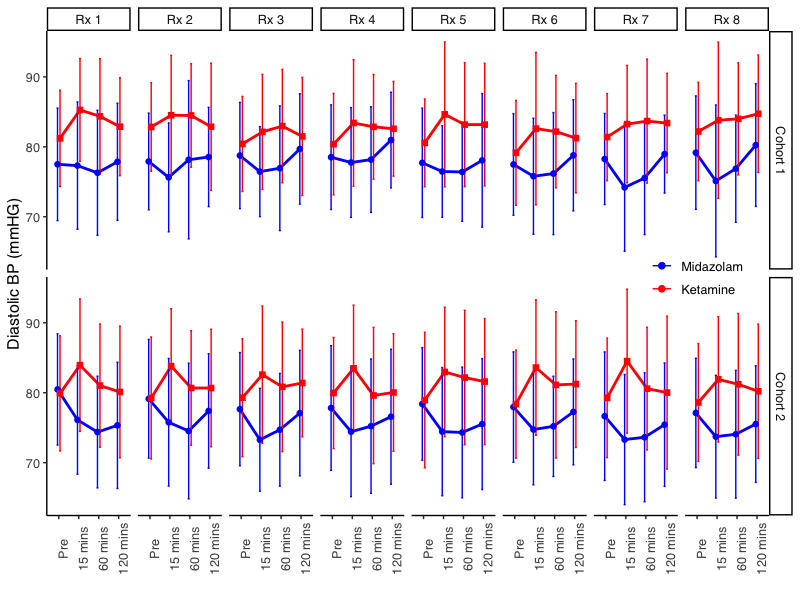

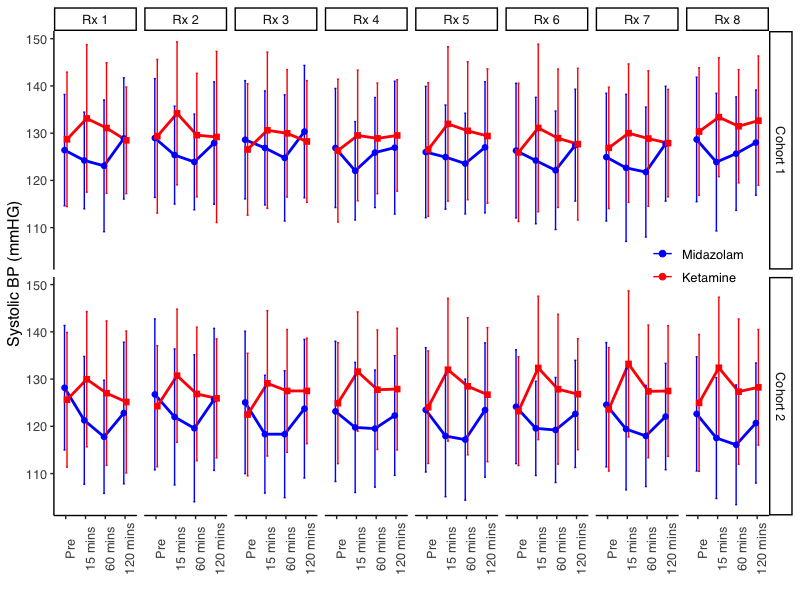


### **Figure S7: Mean changes in heart rate across treatment sessions**

Heart rate measurements, in beats per minute (BPM), were taken pre-injection, and at 15 minutes, 60 minutes and 120 minutes post injection at each treatment session.


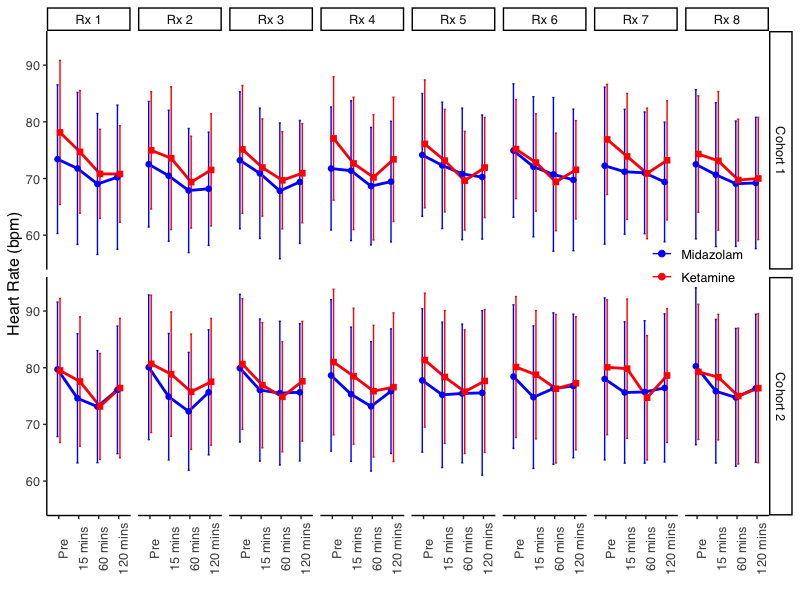


### Table S5: Post-Treatment Adverse Events between treatment sessions.

|  | **Cohort 1** | | | | |  | **Cohort 2** | | | | |
| --- | --- | --- | --- | --- | --- | --- | --- | --- | --- | --- | --- |
|  | **Midazolam**  **(n=35)** | | **Ketamine**  **(n=32)** | |  |  | **Midazolam**    **(n=53)** | | **Ketamine**   **(n=53)** | |  |
| **Adverse Event** | **n** | **(%)** | **n** | **(%)** | **p-value** |  | **n** | **(%)** | **n** | **(%)** | **p-value** |
| Anxiety | 17 | (48.6) | 17 | (53.1) | 0.81 |  | 30 | (56.6) | 28 | (52.8) | 0.85 |
| Moodiness | 19 | (54.3) | 18 | (56.3) | 1.00 |  | 24 | (45.3) | 31 | (58.5) | 0.24 |
| Altered Sleep | 20 | (57.1) | 15 | (46.9) | 0.47 |  | 26 | (49.1) | 30 | (56.6) | 0.56 |
| Weakness/Fatigue | 19 | (54.3) | 20 | (62.5) | 0.62 |  | 21 | (39.6) | 29 | (54.7) | 0.17 |
| Reduced Concentration | 18 | (51.4) | 16 | (50.0) | 1.00 |  | 22 | (41.5) | 27 | (50.9) | 0.44 |
| Headache | 15 | (42.9) | 16 | (50.0) | 0.63 |  | 16 | (30.2) | 34 | (64.2) | 0.001 |
| Increased Energy | 12 | (34.3) | 15 | (46.9) | 0.33 |  | 15 | (28.3) | 30 | (56.6) | 0.006 |
| Restlessness | 9 | (25.7) | 8 | (25.0) | 1.00 |  | 13 | (24.5) | 19 | (35.8) | 0.29 |
| Altered Dreams | 8 | (22.9) | 9 | (28.1) | 0.78 |  | 13 | (24.5) | 13 | (24.5) | 1.00 |
| Diarrhoea | 8 | (22.9) | 6 | (18.8) | 0.77 |  | 12 | (22.6) | 13 | (24.5) | 1.00 |
| Urination (frequency, pain, discomfort) | 6 | (17.1) | 4 | (12.5) | 0.74 |  | 9 | (17.0) | 13 | (24.5) | 0.47 |
| Skin Changes (rash, itch) | 7 | (20.0) | 8 | (25.0) | 0.77 |  | 5 | (9.4) | 9 | (17.0) | 0.39 |
| Constipation | 4 | (11.4) | 5 | (15.6) | 0.73 |  | 5 | (9.4) | 10 | (18.9) | 0.26 |
| Tinnitus | 3 | (8.6) | 6 | (18.8) | 0.29 |  | 4 | (7.5) | 10 | (18.9) | 0.15 |
| Nausea/Vomiting | 3 | (8.6) | 6 | (18.8) | 0.29 |  | 2 | (3.8) | 2 | (3.8) | 1.00 |
| Feeling hot or cold | 0 | (0.0) | 3 | (9.4) | 0.10 |  | 1 | (1.9) | 3 | (5.7) | 0.62 |
| Abdominal pain/Cramps | 0 | (0.0) | 2 | (6.3) | 0.22 |  | 1 | (1.9) | 3 | (5.7) | 0.62 |
| Dissociation | 2 | (5.7) | 2 | (6.3) | 1.00 |  | 2 | (3.8) | 0 | (0.0) | 0.50 |
| Dysgeusia | 1 | (2.9) | 2 | (6.3) | 0.60 |  | 2 | (3.8) | 1 | (1.9) | 1.00 |
| Dizziness | 0 | (0.0) | 3 | (9.4) | 0.10 |  | 0 | (0.0) | 2 | (3.8) | 0.50 |
| Altered/increased perception | 0 | (0.0) | 0 | (0.0) | 1.00 |  | 0 | (0.0) | 3 | (5.7) | 0.24 |

The K-SET Section A reflects new onset or exacerbation of symptoms observed since the participant’s last ketamine or midazolam injection. The incidence of participants experiencing each adverse event was compared between groups using a Fisher’s exact test. Events are reported in order from highest to lowest frequency across all participants.

## Assessment of Blinding

For Cohort 1, for those allocated midazolam, the number correctly guessing treatment allocation at End RCT was 22/33 (66.7%) for participants and 25/33 (75.8%) for raters; for ketamine it was 14/31 (45.2%) for participants and 11/31 (35.5%) for raters. For Cohort 2, corresponding numbers of correct guesses at End RCT in those allocated midazolam were 42/52 (80.8%) for participants and 41/53 (77.4%) for raters; ketamine, 37/53 (69.8%) for participants and 28/53 (52.8%) for raters.

Blinding was assessed by calculating blinding indices as per Bang et al (BBI)(9). The index is scaled to an interval of -1 to 1, 1 being complete lack of blinding, 0 being consistent with perfect blinding and -1 indicating opposite guessing. Scores are calculated separately for each treatment group. Statistically significant unblinding is indicated in bolded values. The values of BBI are interpretable as the proportion who correctly guess treatment arm above (or below) what would be expected by chance.

### Table S6: Bang Blinding Index (BBI) for rater and participant, at End RCT, by cohort and treatment group.

|  | **Cohort 1 (Fixed-dose)** | | | | **Cohort 2 (Flexible-dose)** | | | |
| --- | --- | --- | --- | --- | --- | --- | --- | --- |
|  | **Midazolam** | | **Ketamine** | | **Midazolam** | | **Ketamine** | |
|  | **Est.** | **95% CI** | **Est.** | **95% CI** | **Est.** | **95% CI** | **Est.** | **95% CI** |
| Rater | **0.52** | **0.22 to 0.81*** | -0.29 | -0.63 to 0.05 | **0.55** | **0.32 to 0.78*** | 0.06 | -0.21 to 0.33 |
| Participant | **0.33** | **0.01 to 0.66*** | -0.10 | -0.45 to 0.25 | **0.62** | **0.40 to 0.83*** | **0.40** | **0.15 to 0.64*** |

### Table S7: Stated reasons for allocation guess, at End RCT, by cohort and treatment group

|  | **Cohort 1 (Fixed-dose)** | | | | **Cohort 2 (Flexible-dose)** | | | |
| --- | --- | --- | --- | --- | --- | --- | --- | --- |
|  | **Midazolam**  (n=34) | | **Ketamine**  (n=32) | | **Midazolam**  (n=49) | | **Ketamine**  (n=51) | |
| **Rater** | **n** | **(%)** | **n** | **(%)** | **n** | **(%)** | **n** | **(%)** |
| Efficacy related | 34 | (100.0) | 30 | (93.8) | 44 | (89.8) | 46 | (90.2) |
| Treatment/subjective experience | 0 | (0.0) | 1 | (3.1) | 0 | (0.0) | 2 | (3.9) |
| Both efficacy & treatment experience | 0 | (0.0) | 0 | (0.0) | 3 | (6.1) | 1 | (2.0) |
| Other/non-specific | 0 | (0.0) | 0 | (0.0) | 0 | (0.0) | 0 | (0.0) |
| Unable to give reason/no response provided | 0 | (0.0) | 1 | (3.1) | 2 | (4.1) | 2 | (3.9) |
|  |  | |  | |  | |  | |
| **Participant** | **n** | **(%)** | **n** | **(%)** | **n** | **(%)** | **n** | **(%)** |
| Efficacy related | 19 | (55.9) | 11 | (34.4) | 19 | (38.8) | 16 | (31.4) |
| Treatment/subjective experience | 6 | (17.6) | 12 | (37.5) | 13 | (26.5) | 16 | (31.4) |
| Both efficacy & treatment experience | 8 | (23.5) | 8 | (25.0) | 12 | (24.5) | 13 | (25.5) |
| Other/non-specific | 1 | (2.9) | 1 | (3.1) | 3 | (6.1) | 4 | (7.8) |
| Unable to give reason/no reason provided | 0 | (0.0) | 0 | (0.0) | 2 | (4.1) | 2 | (3.9) |

# References

1. Short B, Dong V, Galvez V, et al: Development of the Ketamine Side Effect Tool (KSET). J Affect Disord 2020; 266:615-620

2. Posner K, Brown GK, Stanley B, et al: The Columbia–Suicide Severity Rating Scale: Initial Validity and Internal Consistency Findings From Three Multisite Studies With Adolescents and Adults. Am J Psychiatry 2011; 168:1266-1277

3. Bremner JD, Krystal JH, Putnam FW, et al: Measurement of dissociative states with the Clinician-Administered Dissociative States Scale (CADSS). J Traum Stress 1998; 11:125-136

4. Overall JE, Gorham DR: The Brief Psychiatric Rating Scale. Psychological reports 1962; 10:799

5. Young RC, Biggs JT, Ziegler VE, et al: A Rating Scale for Mania: Reliability, Validity and Sensitivity. Br J Psychiatry 1978; 133:429-435

6. Humphrey L, Arbuckle R, Moldwin R, et al: The bladder pain/interstitial cystitis symptom score: development, validation, and identification of a cut score. European urology 2012; 61:271-279

7. European Medicines Agency: Guideline on the investigation of subgroups in confirmatory clinical trials 2019; https://www.ema.europa.eu/en/investigation-subgroups-confirmatory-clinical-trials

8. US Food and Drug Administration: Briefing information for the Feb 12, 2019 joint meeting of the Psychopharmacologic Drugs Advisory Committee (PDAC) and the Drug Safety and Risk Management Advisory Committee (DSaRM) 2019; https://www.fda.gov/advisory-committees/february-12-2019-joint-meeting-psychopharmacologic-drugs-advisory-committee-pdac-and-drug-safety-and

9. Bang H, Ni L, Davis CE: Assessment of blinding in clinical trials. Controlled Clinical Trials 2004; 25:143-156
